# Ultrasound‐Responsive 4D Bioscaffold for Synergistic Sonopiezoelectric‐Gaseous Osteosarcoma Therapy and Enhanced Bone Regeneration

**DOI:** 10.1002/advs.202417208

**Published:** 2025-04-03

**Authors:** Haoyu Fang, Daoyu Zhu, Yixuan Chen, Changqing Zhang, Gan Li, Qihang Fang, Meiqi Chang, Yu Chen, Youshui Gao

**Affiliations:** ^1^ Department of Orthopedic Surgery Shanghai Sixth People's Hospital Affiliated to Shanghai Jiao Tong University School of Medicine Shanghai 200233 China; ^2^ Laboratory Center, Shanghai Municipal Hospital of Traditional Chinese Medicine Shanghai University of Traditional Chinese Medicine Shanghai 200071 China; ^3^ Materdicine Lab, School of Life Sciences Shanghai University Shanghai 200444 China

**Keywords:** black phosphorus, bone regeneration, NO generation, sonopiezoelectric effect, tumor eradication

## Abstract

Various antitumor strategies have emerged to address the escalating need for effective tumor eradication. However, achieving precise and spatiotemporally controlled dynamic therapies remains promising yet challenging. Sonopiezoelectric nanotherapy eliminates tumor cells by generating reactive oxygen species (ROS) through ultrasound stimulation, enabling spatiotemporal control and ensuring safety during deep tissue penetration. In this study, a hybrid bioscaffold incorporating few‐layer black phosphorus (BP) and nitric oxide (NO) donors are rationally designed and engineered for sonopiezoelectric‐gaseous synergistic therapy. This ultrasound‐responsive system provides a stepwise countermeasure against tumor invasion in bone tissues. Ultrasonic vibration induces mechanical strain in BP nanosheets, leading to piezoelectric polarization and subsequent ROS generation. Moreover, ultrasound‐triggered NO burst release from the donors enables spatiotemporally controlled gas therapy. The synergistic effects of sonopiezoelectric therapy and ultrasound‐excited gas therapy enhance tumor eradication, effectively inhibiting tumor proliferation and metastasis while minimizing off‐target cytotoxicity. Additionally, the biomineralization capability of degradable BP and proangiogenic effects of low‐concentration NO establish the hybrid bioscaffold as a bioactive platform that facilitates subsequent bone regeneration. The development of this 4D multifunctional therapeutic platform, characterized by superior sonopiezoelectric efficacy, controlled NO release, and stimulatory effects on tissue regeneration, offers new insights into the comprehensive treatment of invasive bone tumors.

## Graphical abstract

An acoustic‐responsive therapeutic platform for synergistic sonopiezoelectric‐gaseous osteosarcoma therapy and bone regeneration is fabricated through the joint loading of few‐layer black phosphorus nanosheets and S‐Nitroso‐l‐glutathione onto the 3D‐printed bioactive glass scaffolds. The strategic incorporation of sonodynamic therapy, gas therapy, and in situ tissue regeneration therapy may emerge as a spatiotemporally controllable stepwise countermeasure against invasive bone tumors.

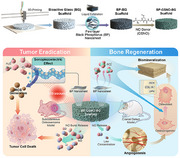

## 1 Introduction

The drawbacks inherent to traditional surgical treatment of bone tumors, coupled with the high recurrence rate of refractory osteosarcoma, have necessitated the development of multifunctional therapeutic platforms for spatiotemporally controlled tumor eradication and subsequent bone regeneration.^[^ ^1^ ^]^ In recent decades, various dynamic therapies have emerged as promising antitumor strategies to address the escalating need for osteosarcoma treatment. These therapies are characterized by their high efficacy, excellent specificity, and mitigated off‐target effects.^[^ ^2^ ^]^ A key feature of dynamic therapies is their ability to convert exogenous energy (e.g., near‐infrared light, X‐rays, electric currents, ultrasound) or endogenous energy (e.g., biochemical reactions within the tumor microenvironment) into the production of reactive oxygen species (ROS), which induce tumor cell death by disrupting critical cellular components essential for tumor survival and proliferation.^[^ ^3^ ^]^ Based on their excitation activator, dynamic therapeutic strategies can be broadly classified into photodynamic,^[^ ^2^, ^3^, ^4^ ^]^ radiodynamic,^[^ ^5^ ^]^ chemodynamic,^[^ ^6^ ^]^ electrodynamic,^[^ ^7^ ^]^ and sonodynamic therapies.^[^ ^8^ ^]^ Among them, sonopiezoelectric therapy, an emerging non‐invasive dynamic therapeutic approach utilizing ultrasound as the exogenous energy source, offers precise spatiotemporal control and superior tissue penetration depth, making it particularly advantageous for the treatment of deep‐seated tumors. Ultrasound (US), as a non‐invasive modality, has been widely employed in clinical applications, including diagnostic imaging, US‐assisted drug delivery, controlled drug release, and ultrasonic tumor theranostics.^[^ ^9^ ^]^ Due to its non‐ionizing nature and low tissue attenuation coefficient, ultrasound can penetrate deep tissue layers while minimizing damage to surrounding healthy tissues, establishing it as one of the safest theranostic options. Despite these well‐recognized advantages, the clinical translation of sonopiezoelectric therapy remains limited due to the scarcity of suitable piezoelectric materials, which are required for excellent stability under ultrasonic stimulation and superior biodegradability to prevent cumulative toxicity. Addressing these challenges requires the rational design of novel piezoelectric biomaterials that balance therapeutic efficacy with biocompatibility, ensuring their potential for clinical applications in osteosarcoma treatment and bone regeneration.

As a piezoelectric material, few‐layer black phosphorus (BP) nanosheets exhibit superior piezoelectricity, excellent biocompatibility, and satisfactory biodegradability, making them an ideal sonosensitizer for sonopiezoelectric therapy. Piezoelectricity, a property that has garnered increasing attention, refers to the ability to convert ambient mechanical energy (e.g., ultrasonic vibrations) into an intrinsic electric field and vice versa, which is dubbed the piezoelectric effect.^[^ ^10^ ^]^ Due to its non‐centrosymmetric lattice structure, few‐layer BP nanosheets with a layer‐dependent tunable band gap demonstrate remarkable piezoelectric properties.^[^ ^10^, ^11^ ^]^ Upon ultrasonic vibration, the piezoelectric polarization of few‐layer BP nanosheets induces band bending, shifting the conduction band edge to a potential more negative than the O_2_/O_2_^•−^ redox potential and the valence band edge to a potential more positive than the H_2_O/·OH redox potential, thereby facilitating reactive oxygen species (ROS) generation.^[^ ^10^, ^11^ ^]^ Additionally, few‐layer BP nanosheets maintain structural stability within the duration of ultrasonic stimulation but gradually degrade into biocompatible phosphorus‐based products over time, supporting their potential for in vivo clinical applications.^[^ ^2^, ^11^, ^12^ ^]^ Excess ROS generated through the ultrasound‐induced piezocatalytic process further accelerates this degradation behavior.^[^ ^11c^ ^]^ The degradation products of BP nanosheets include non‐toxic phosphate ions (PO_4_^3−^), which are the key component of the skeletal system for promoting osteogenesis and osseointegration.

Nitric oxide (NO), an endogenous gaseous signaling molecule, plays a pivotal role in regulating various physiological processes, including cell proliferation, vasodilation, angiogenesis, inflammation modulation, neurotransmission, and extracellular matrix deposition. Due to its broad physiological relevance, NO holds significant potential for diverse clinical applications, including tumor therapy, tissue regeneration, cardiovascular treatment, and antimicrobial strategies.^[^ ^13^ ^]^ The therapeutic functionalities of NO are primarily dependent on its local concentration. Specifically, at high concentrations, NO not only compromises mitochondrial membrane permeability by inducing cytochrome C release but also initiates p53 phosphorylation and acetylation (at concentrations exceeding ≈400 nmol L^−1^), leading to cell cycle arrest, apoptosis, and necrosis.^[^ ^13d,i^ ^]^ Moreover, further elevation of NO levels (above ≈1 µmol L^−1^) induces the formation of reactive nitrogen species such as dinitrogen trioxide (N_2_O_3_) and peroxynitrite (ONOO⁻), which exert potent nitrosative stress by inducing DNA damage through deamination and blocking DNA repair via nitrosation of DNA alkyltransferase.^[^ ^13^, ^14^ ^]^ Given its potent antitumor effect, ephemeral half‐life (≈5 s), and limited diffusion radius (≈ranging from 40 to 200 µm), NO enables NO‐based tumor therapies to achieve efficient tumor eradication in a spatiotemporally controlled manner with minimal off‐target effects on healthy tissues.^[^ ^15^ ^]^ Additionally, at low concentrations (below ≈50 nmol L^−1^), NO acts as a proangiogenic regulator, promoting endothelial cell proliferation and migration through activation of the cyclic guanosine 3′,5′‐monophosphate (cGMP)/extracellular signal‐regulated kinase (ERK) signaling pathway.^[^ ^13^, ^16^ ^]^ In addition to stimulating neovascularization during bone regeneration, the sustained release of low‐dose NO enhances cell proliferation, osteogenic differentiation of osteoblasts, collagen deposition, and calcium ion mobilization for biomineralization, thereby accelerating osteogenesis and osseointegration.^[^ ^16^, ^17^ ^]^ Consequently, efficient tumor eradication can be achieved through the localized administration of high‐concentration NO over a short period, whereas the prolonged release of low‐concentration NO functions as a proangiogenic and osteogenic modulator to promote osteogenesis and osseointegration. This dual therapeutic role establishes NO as a promising candidate for osteosarcoma treatment and subsequent bone repair.

**Scheme 1:**
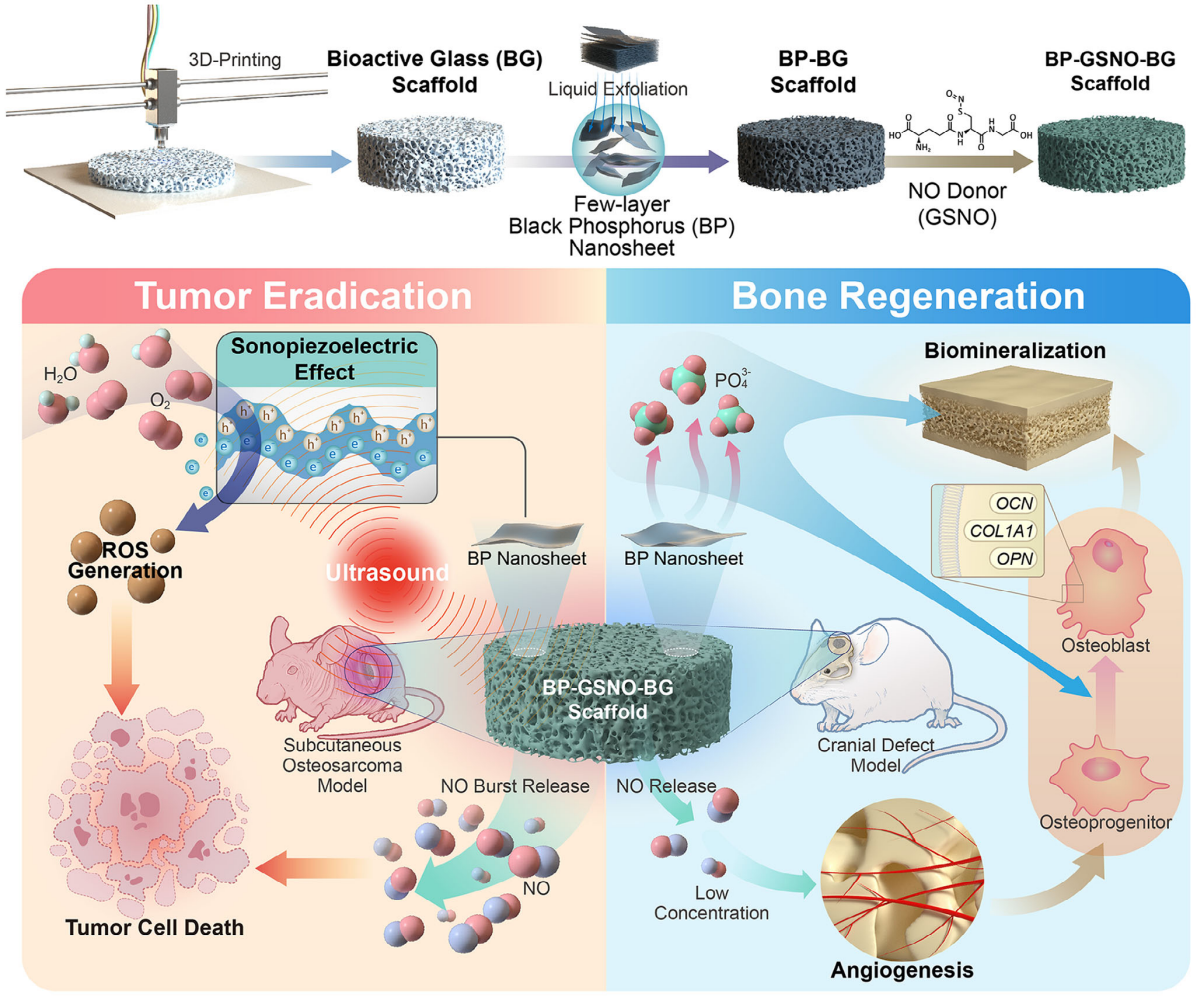
Schematic representation of the fabricated ultrasound‐responsive BP‐GSNO‐BG scaffold for combinatory osteosarcoma sonopiezoelectric therapy and bone regeneration.

Herein, we present a novel acoustically responsive 4D biomaterial (BP‐GSNO‐BG) for synergistic osteosarcoma therapy and bone regeneration through the joint loading of few‐layer BP nanosheets and S‐nitroso‐L‐glutathione (GSNO, a biocompatible nitric oxide (NO) donor with excellent NO‐releasing capability^[^ ^16^, ^18^ ^]^) onto a 3D‐printed bioactive glass (BG) scaffold (**Scheme** **1**). The phenomenal biocompatibility, osteoconductivity, and osteoinductivity of bioactive glass scaffolds have been widely acknowledged and extensively documented in the literature.^[^ ^19^ ^]^ The anticancer performance of the BP‐GSNO‐BG scaffold is enabled by ultrasound‐activated piezoelectric ROS generation from few‐layer BP nanosheets, accompanied by the ultrasound‐triggered burst release of high‐concentration NO from GSNO. Furthermore, the in situ biodegradation of BP nanosheets produces non‐toxic phosphate ions (PO_4_^3−^) ions, which facilitate osteogenesis and osseointegration. At a later stage, the gradual release of low‐concentration NO functions as both a proangiogenic regulator and an osteogenic factor, synergistically accelerating bone regeneration. The remarkable multifunctionalities of this hybrid bioscaffold, including enhanced anticancer activity and superior bone regeneration capability, have been systematically investigated through comprehensive in vitro and in vivo evaluations. Additionally, integrating the concept of time as the fourth dimension, 4D biomaterials undergo dynamic shape‐transformation or functional‐transformation in response to predefined stimuli, enabling precise control over the hierarchical architecture and functionality of biomimetic tissue surrogates.^[^ ^19^, ^20^ ^]^ This multifunctional therapeutic platform exhibited functional transformation throughout the treatment course, transitioning from an antitumor role to an osteogenic function. This exceptional capability aligns with the emerging paradigm of 4D biomaterials, offering a stepwise countermeasure against tumor invasion in bone tissues.

## 2 Results and Discussion

### 2.1 Fabrication and Characterization of Hybrid Scaffolds

**Figure 1:**
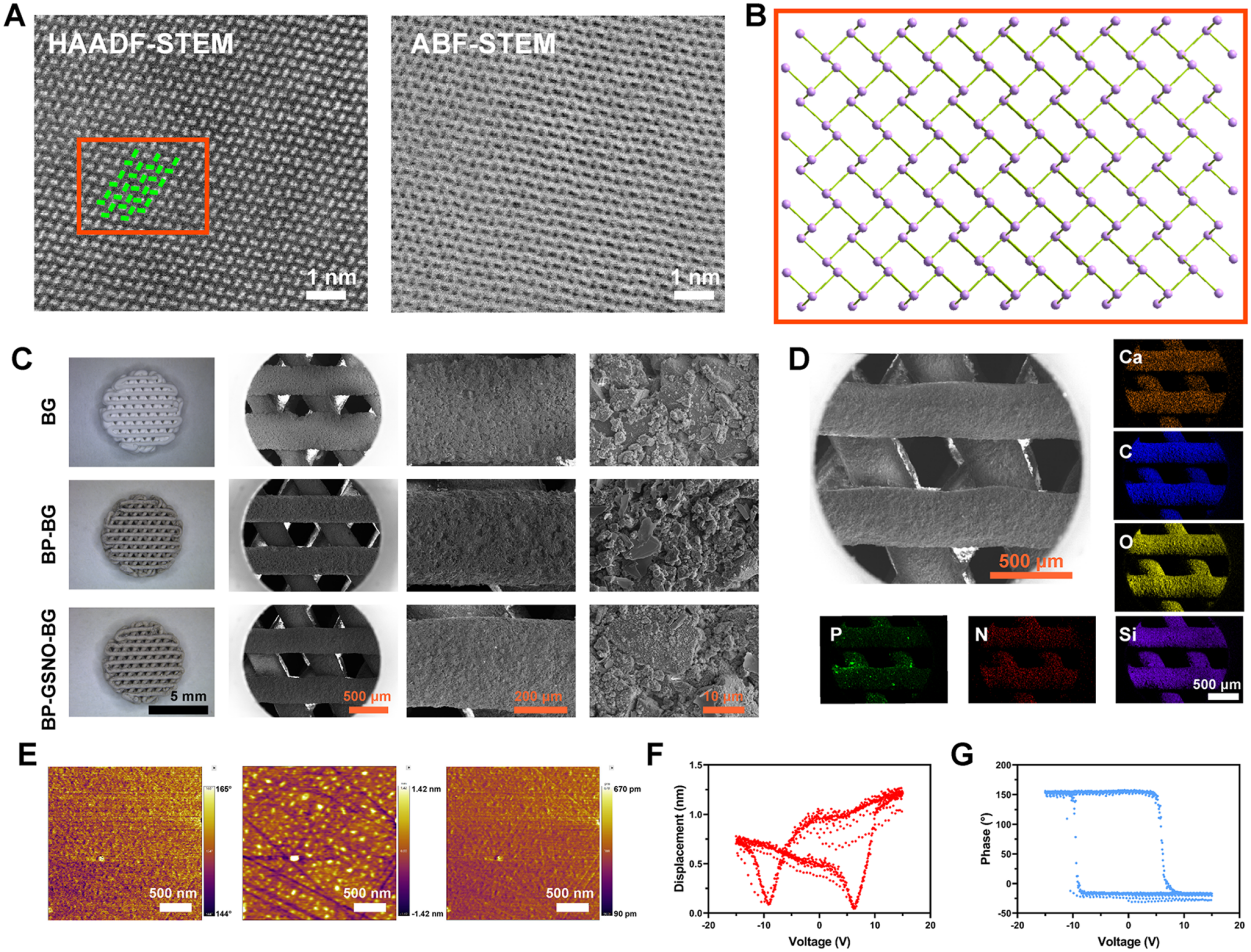
Characterization of BP nanosheets and the hybrid scaffolds. A) HAADF‐STEM image and ABF‐STEM image of BP nanosheets (Scale bar: 1 nm). B) Ball‐and‐stick model of BP nanosheets. C) Digital photographs and scanning electron microscopy (SEM) images of different scaffolds with 3D hierarchical structure. Images of different scaffolds at various magnifications, arranged from left to right. D) Element mappings of the BP‐GSNO‐BG scaffold (Scale bar: 500 µm). E) Piezoresponse force microscopy (PFM) phase image and amplitude image of BP nanosheets at the tip‐substrate voltage of 15 V (Scale bar: 500 nm). F) Hysteresis loops under different tip‐substrate voltages in an amplitude signal channel. G) Hysteresis loops under different tip‐substrate voltages in phase signal channel.

The BP‐GSNO‐BG scaffolds were fabricated as a multifunctional therapeutic platform by integrating few‐layer BP nanosheets and GSNO, an NO donor, into the BG scaffolds via the surface modification technique. The morphology of the 2D few‐layer BP nanosheets was characterized using transmission electron microscopy (TEM), which demonstrated a uniform lateral size distribution (Figures S1 and S2, Supporting Information). High‐angle annular dark‐field scanning transmission electron microscopy (HAADF‐STEM) and annular bright‐field scanning transmission electron microscopy (ABF‐STEM) further revealed uniformly aligned lattice fringes of the few‐layer BP nanosheets (**Figure** **1**A), as illustrated by the ball‐and‐stick model in Figure 1B. Given that 3D microenvironments more effectively support key cellular activities, such as adhesion, proliferation, and differentiation, compared to 2D topographical biomaterials, BG scaffolds were employed as the structural foundation of the multifunctional platform. The BG scaffolds were fabricated using a precisely controlled 3D printing technique, and their geometrical structure was thoroughly analyzed. The matte surface topography and hierarchical porous structure—comprising interconnected macropores and micropores—of BP‐GSNO‐BG scaffolds promote biomolecule adsorption, cellular adhesion, migration, differentiation, neovascularization, and biomineralization, thereby facilitating osteogenesis and osseointegration^[^ ^19b^ ^]^ (Figure 1C). Following surface modification with BP nanosheets, the BG scaffolds exhibited a color transition from white to light black, with scanning electron microscopy (SEM) images confirming the presence of BP nanosheets on the roughened surface of the hybrid scaffolds. Element mapping of the BP‐GSNO‐BG scaffolds revealed a homogeneous distribution of phosphorus (P), nitrogen (N), silicon (Si), oxygen (O), carbon (C), and calcium (Ca), confirming the successful surface functionalization with few‐layer BP nanosheets and NO donors (Figure 1D). Notably, the effective functionalization of BP nanosheets and NO donors onto the BG scaffolds can be attributed to the unique topographical morphology and hierarchical porous structure of the scaffolds. Furthermore, to prevent premature biodegradation of BP nanosheets, the functionalization process was conducted in a water‐free environment, delaying degradation until the scaffolds were implanted in vivo.

The superior piezoelectricity of BP nanosheets originates from their non‐centrosymmetric crystal structure.^[^ ^10^, ^11^ ^]^ Under mechanical stress, transient deformation of the highly ordered crystal lattice induces atomic displacement, resulting in charge accumulation via an ordered dipole distribution.^[^ ^10f^ ^]^ The piezoelectric effect of few‐layer BP nanosheets was verified using piezoresponse force microscopy (PFM) (Figure 1E). The topographical image of BP nanosheets revealed the characteristic morphology of a 2D nanosheet, with distinct contrasts observed in both amplitude and phase images. Upon application of a localized exogenous electric field through the PFM tip, hysteresis loops appeared in both amplitude and phase signal channels, exhibiting a ≈180° phase shift corresponding to the reversal of the applied voltage (Figure 1F,G), thereby confirming the pronounced piezoelectric properties of few‐layer BP nanosheets. These findings align with previous reports on the piezoelectricity of BP nanosheets.

**Figure 2:**
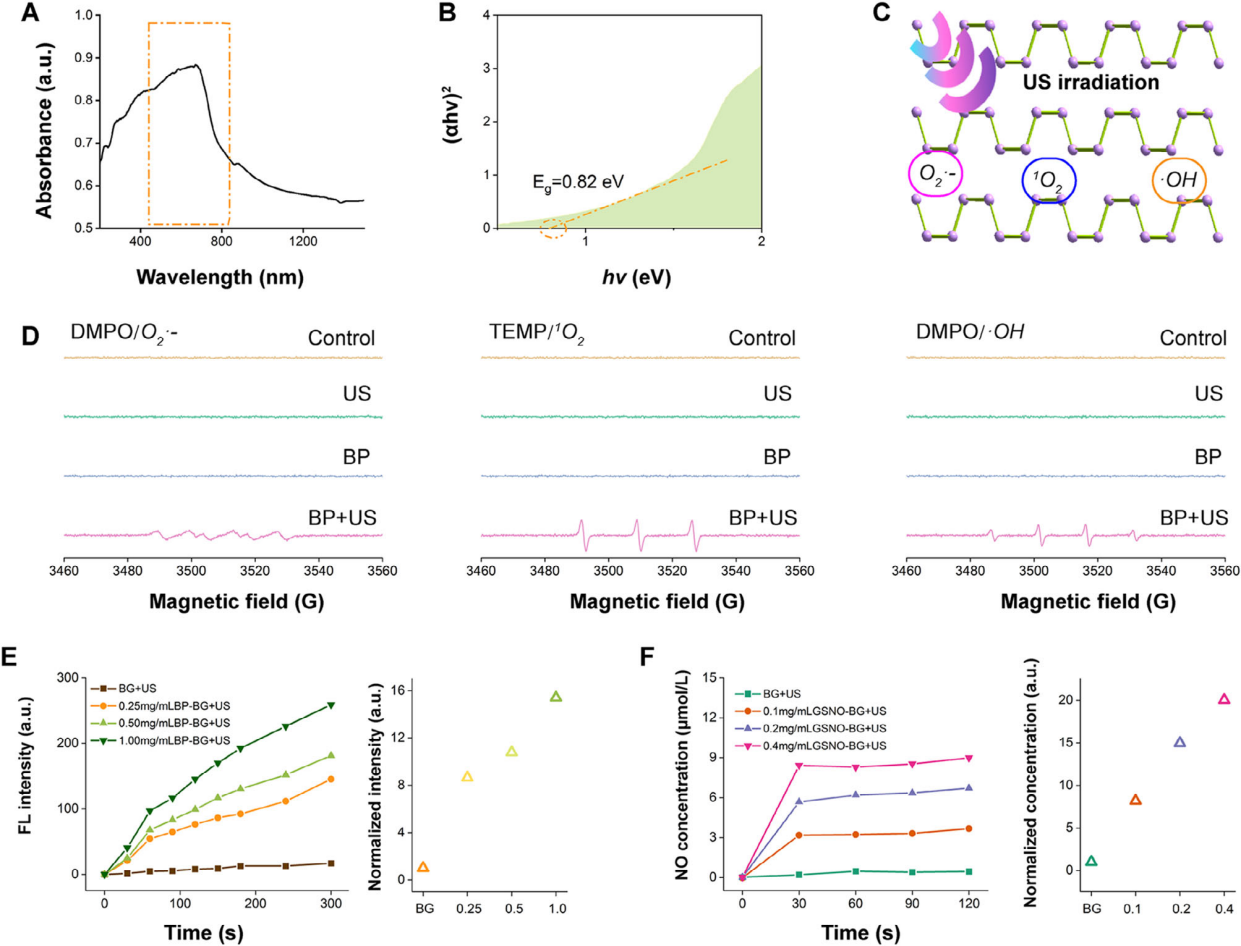
Piezoelectric effect and ROS generating capability of the hybrid nanosheets. (A) UV–Vis‐NIR diffuse reflection spectroscopy of the BP nanosheets. (B) Tauc plot of the BP nanosheets. (C) Illustration of the sonopiezoelectric effect of BP nanosheets. Under ultrasonic irradiation, BP nanosheet dispersions could generate diverse ROS, including ·OH, ^1^O_2_, and O_2_^•−^. (D) Electron spin resonance (ESR) spectra of DMPO/O_2_^•−^, TEMP/^1^O_2_, and DMPO/·OH in BP nanosheet dispersions with ultrasonic stimulation (1 MHz, 1.2 W/cm^2^ for 60 s) (referred to as the “BP + US” group), with those of BP nanosheet dispersions without ultrasonic stimulation (referred to as the “BP” group) included as a reference. (E) ROS release efficiency of BP‐BG scaffolds under ultrasonic stimulation. (F) NO release efficiency of GSNO‐BG hybrid scaffolds under ultrasonic stimulation.

The absorption characteristics of BP nanosheets and their bandgap of 0.82 eV were confirmed (**Figure** **2**A,B), demonstrating the electronic transport properties of BP. To verify the ultrasound‐induced ROS‐generating capability of few‐layer BP nanosheets, electron spin resonance (ESR) spectra of BP nanosheet dispersions were monitored. The results confirmed that BP nanosheets generated hydroxyl radicals (·OH), singlet oxygen (^1^O_2_), and superoxide anions (O_2_^•−^) under ultrasonic stimulation (1 MHz, 1.2 W cm^−2^ for 60 s) (Figure 2C,D). The ROS release profile of BP‐BG scaffolds and the NO release pattern of GSNO‐BG scaffolds were investigated using a ROS detecting kit and the Griess assay, respectively (Figure 2E,F). These findings further corroborated the robust ROS‐generating and NO‐releasing capabilities of the hybrid scaffolds, which contribute to their cytotoxic effects on tumor cells. Notably, the ROS‐generating capability of BP‐BG scaffolds was positively correlated with the concentration of BP dispersion solution used during scaffold fabrication, while the NO‐releasing capability of GSNO‐BG scaffolds was positively correlated with the concentration of GSNO dispersion solution during scaffold fabrication. Based on these findings, we selected 1.00 mg mL^−1^ BP dispersion solution and 0.40 mg mL^−1^ GSNO dispersion solution as the standardized soaking solutions for the fabrication of BP‐BG scaffolds, GSNO‐BG scaffolds, and BP‐GSNO‐BG hybrid scaffolds. The static NO release profile of the GSNO‐BG scaffold indicated that, in the absence of ultrasound exposure, low‐concentration NO (≈100 nmol L^−1^) was released from the hybrid scaffold, highlighting the proangiogenic potential of NO molecules (Figure S4, Supporting Information). In summary, these results confirmed that BP‐GSNO‐BG hybrid scaffolds efficiently generate diverse ROS and high concentrations of NO gas (>1 µmol L^−1^) under ultrasonic excitation, demonstrating superior cytotoxicity against tumor cells and facilitating tumor eradication.

### 2.2 In Vitro Cell Ablation Effect of Sonopiezoelectric Scaffolds

**Figure 3:**
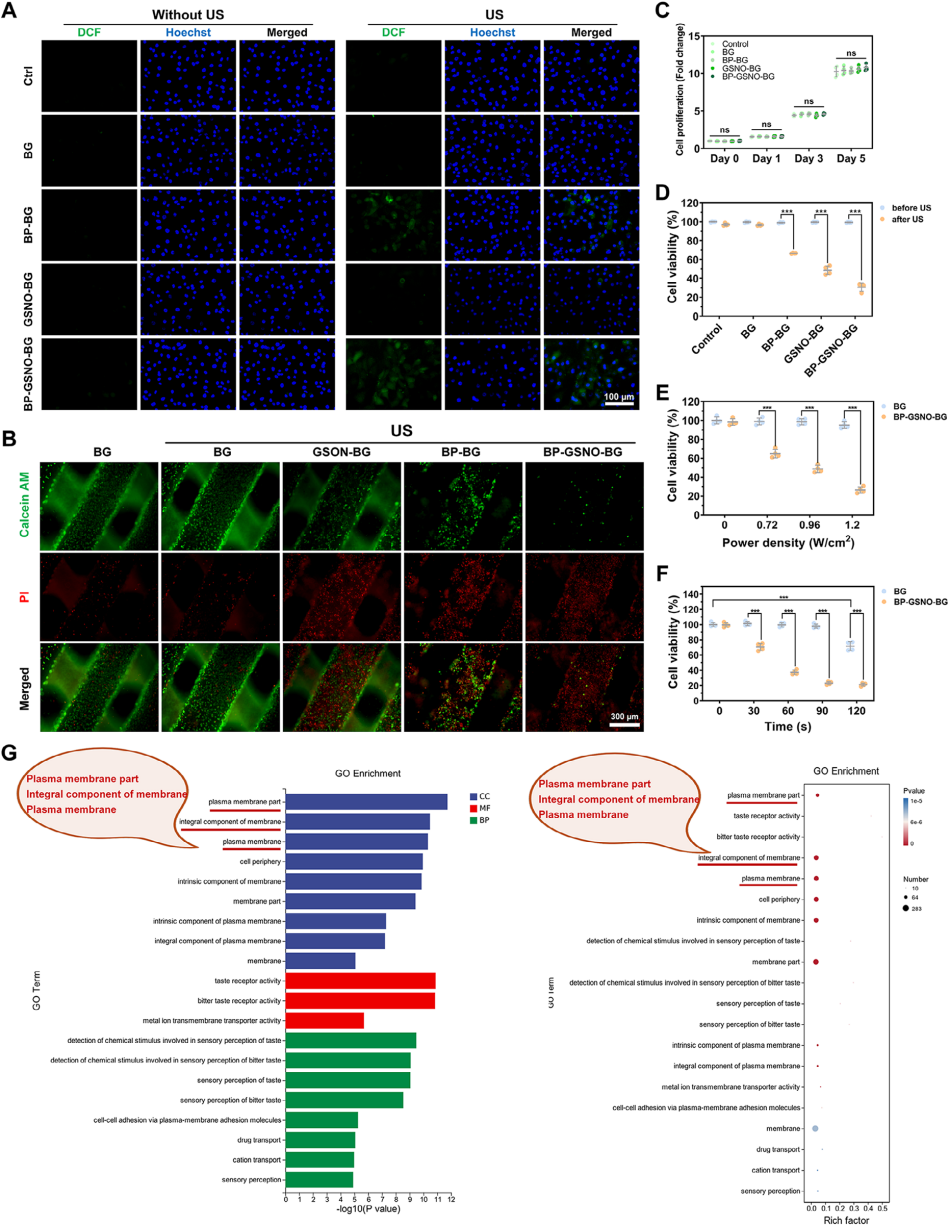
In vitro cell ablation effect of BP‐GSNO‐BG scaffold. A) ROS production efficiency of MG63 cells treated with different groups (Scale bar: 100 µm. US refers to ultrasound stimulation (1 MHz) at 1.2 W/cm^2^ for 30 s). B) Live/dead viability assay of MG63 cells treated with different groups. MG63 cells were stained with calcein AM (live cells, green fluorescence) and PI (dead cells, red fluorescence) on different scaffolds with/without ultrasound stimulation (Scale bar: 300 µm. US refers to ultrasound stimulation (1 MHz) at 1.2 W/cm^2^ for 90 s). C) CCK‐8 cell proliferation assay of MG63 cells under different culture conditions (Statistics were derived using the two‐way analysis of variance. All data were expressed as mean ± standard deviation (SD). *n* = 4 for each group; ns, no significance). D) CCK‐8 cell viability assay of MG63 cells before/after ultrasonic stimulation. MG63 cells were co‐cultured with different scaffolds for 1 day before treatment (Statistics were derived using the two‐way analysis of variance. All data were expressed as mean ± standard deviation (SD). *n* = 4 for each group, ^***^ *p* < 0.001. US refers to ultrasound stimulation (1 MHz) at 1.2 W/cm^2^ for 90 s). E,F) CCK‐8 cell viability assay of MG63 cells at various ultrasound power densities and duration time. MG63 cells were co‐cultured with different scaffolds for 1 day before treatment (Statistics were derived using the two‐way analysis of variance. All data were expressed as mean ± standard deviation (SD). *n* = 4 for each group, ^***^ *p* < 0.001). G) The Gene Ontology (GO) enrichment plots of MG63 cells under different treatment (Treatment group: MG63 cells co‐cultured with the BP‐GSNO‐BG scaffold for 24 h and stimulated by US (1 MHz) at 1.2 W/cm^2^ for 30 s. Ctrl group: MG63 cells cultured under normal conditions and stimulated by the US (1 MHz) at 1.2 W/cm^2^ for 30 s).

To evaluate the sonopiezoelectric therapeutic effect at the cellular level, ROS generation by BP‐GSNO‐BG bioscaffolds with and without ultrasonic stimulation was first quantified using dichlorodihydrofluorescein diacetate (DCFH‐DA) as the fluorescent probe. We utilized the transwell chamber system to co‐culture osteosarcoma cells (MG63) with hybrid scaffolds. Under ultrasonic excitation, MG63 cells co‐cultured with BP‐BG and BP‐GSNO‐BG scaffolds exhibited a significant increase in intracellular ROS levels, as evidenced by enhanced fluorescence intensity in the cytoplasm (**Figure** **3**A). Concurrently, MG63 cells co‐cultured with GSNO‐BG and BP‐GSNO‐BG scaffolds demonstrated substantial NO release under ultrasonic excitation, as detected by the DAF‐FM DA fluorescence probe (Figure S5, Supporting Information). Fluorescence imaging from the live/dead cell viability assay revealed distinct fluorescence emission patterns corresponding to live and dead cells. These images not only highlighted a clear contrast before and after ultrasonic stimulation, confirming the ultrasound‐induced cytotoxicity of BP‐GSNO‐BG scaffolds but also provided insights into the intricate hierarchical porous architecture of the hybrid scaffolds (Figure 3B). Notably, osteosarcoma cells exhibited strong adhesion and proliferation on the hybrid scaffolds during the 5‐day co‐culture period in the absence of ultrasound stimulation, indicating the excellent cytocompatibility of the scaffolds (Figure 3C).

Furthermore, the viability of MG63 cells co‐cultured with BP‐BG, GSNO‐BG, and BP‐GSNO‐BG scaffolds was significantly reduced following ultrasound exposure compared to the BG group (Figure 3D), suggesting the superior tumor‐eradication efficacy of BP‐GSNO‐BG scaffolds at the cellular level and demonstrating their vast feasibility for in vivo antitumor applications. Comparatively, the effects of ultrasound power density and exposure duration on MG63 cell eradication efficiency were systematically evaluated to determine the optimal parameters for tumor eradication and assess the clinical applicability of BP‐GSNO‐BG scaffolds (Figure 3E,F). The results indicated that higher power densities and longer exposure times led to enhanced tumor eradication efficacy. Specifically, a power density of 1.2 W cm^−2^ and exposure duration of 90 s were identified as the optimal parameters for effectively eradicating MG63 osteosarcoma cells. Additionally, high‐throughput analyses were conducted to thoroughly investigate the underlying mechanism of ultrasound‐induced cytotoxicity of BP‐GSNO‐BG scaffolds (Figures S6 and S7, Supporting Information). Gene Ontology (GO) enrichment analysis and Gene Set Enrichment Analysis (GSEA) revealed that the combinatorial tumor cell eradication effect of the BP‐GSNO‐BG hybrids scaffold involves interference with and disruption of tumor cell membrane integrity (Figure 3G). This effect also influences chemical stimulus detection pathways (Figure S8, Supporting Information).

### 2.3 In Vivo Tumor Ablation Effect of Sonopiezoelectric Scaffolds

**Figure 4:**
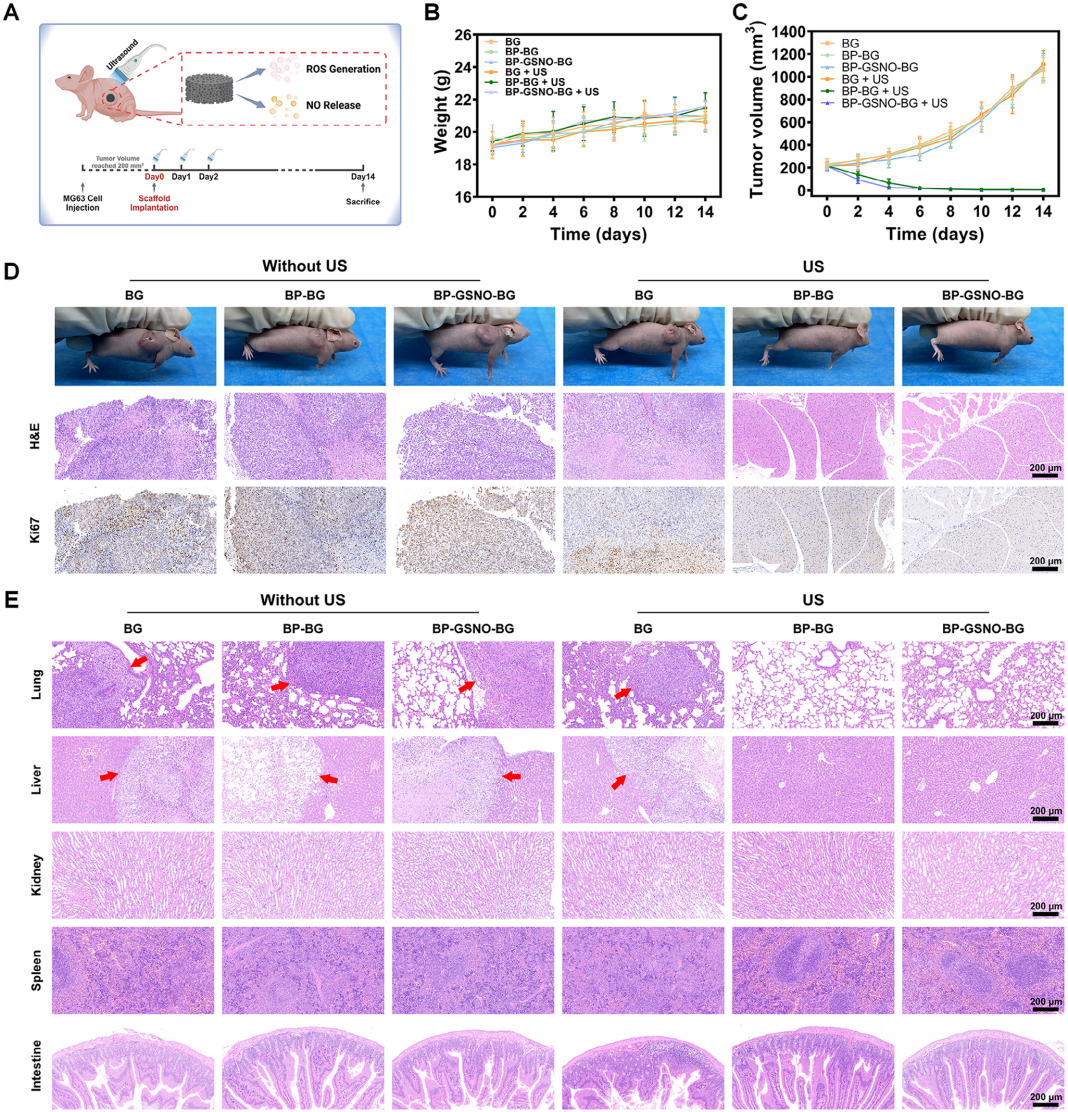
In vivo tumor ablation effect of BP‐GSNO‐BG scaffold. A) Schematic illustration of BP‐GSNO‐BG scaffolds for in vivo subcutaneous tumor ablation. Created in BioRender. Fang, H. (2025). B) Time‐dependent body‐weight curves after different treatments (All data were expressed as mean ± standard deviation (SD). *n* = 5 for each group). C) Time‐dependent tumor‐growth curves after different treatments (All data were expressed as mean ± standard deviation (SD). *n* = 5 for each group). D) Digital photographs of subcutaneously osteosarcoma‐bearing mice following different treatments on day 14, along with the H&E and Ki‐67 staining of the tumor tissues (Scale bar, 200 µm). E) H&E staining of major organs (lung, liver, spleen, kidney, and intestine) of the mice after different treatments on day 14. Red arrows indicate for the metastatic tumors in major organs (Scale bar, 200 µm).

The therapeutic efficacy of ultrasound‐triggered sonopiezoelectric‐gaseous tumor therapy was further evaluated using a subcutaneous osteosarcoma model in female BALB/c nude mice by subcutaneously injecting MG63 cell suspension into the axillary region of the mice (**Figure** **4**A). Once tumor volumes reached ≈200 mm^3^, the tumor‐bearing mice were randomly divided into six groups (BG group, BP‐BG group, BP‐GSNO‐BG group, BG + US group, BP‐BG + US group, and BP‐GSNO‐BG + US group) for diverse treatment based on the results of the in vitro experiments. Following surgical implantation of scaffolds at the tumor site, ultrasound exposure (1 MHz, 1.2 W cm^−2^ for 10 min) was administered to the designated groups on day 0, day 1, and day 2.

Body weights and tumor volumes of tumor‐bearing nude mice were meticulously monitored to assess the in vivo biosafety and therapeutic efficacy of the different treatment modalities. Mice in all groups exhibited negligible weight fluctuations with no significant adverse effects, confirming the excellent biosafety and biocompatibility of the proposed treatment strategies (Figure 4B). Notably, tumors in mice from the BP‐BG + US and BP‐GSNO‐BG + US groups were nearly eradicated without any recurrence throughout the 14‐day observation period (Figure 4C,D), with tumor growth inhibition (TGI) values reaching 99.46 ± 0.28% and 99.65 ± 0.24%, respectively (Figure S9, Supporting Information). In contrast, rapid tumor progression in terms of tumor size was observed in mice from the BG, BP‐BG, BP‐GSNO‐BG, and BG + US groups.

The tumor eradication efficacy of each treatment was further evaluated by hematoxylin and eosin (H&E) staining, while in vivo tumor cell proliferation was assessed via immunohistochemical staining of Ki67 (Figure 4D). H&E staining of subcutaneous osteosarcoma tissues revealed that atypical tumor cells were rarely detected in the BP‐BG + US and BP‐GSNO‐BG + US groups compared to the other groups (Figure 4D), which could be attributed to the increased apoptosis and necrosis observed in these two groups. Immunohistochemical staining of Ki67 showed minimal proliferative cancerous cells (stained dark brown) in the BP‐BG + US and BP‐GSNO‐BG + US groups, indicating significant suppression of tumor cell proliferation. To evaluate the off‐target effects and histocompatibility of these therapeutic modalities, H&E staining of mouse major organs (lung, liver, spleen, kidney, and intestine) was performed after the 14‐day treatment period. No evident pathological toxicities were observed, demonstrating the superior histocompatibility of the hybrid scaffolds (Figure 4E). Metastatic tumors were frequently observed in lung and liver tissues across multiple groups, whereas no metastatic tumors were detected in the major organs of mice in the BP‐BG + US and BP‐GSNO‐BG + US groups, strongly confirming the exceptional therapeutic efficacy of these treatments in eradicating tumors and inhibiting metastasis. These in vivo experimental results demonstrate that the BP‐GSNO‐BG hybrid scaffold exhibits excellent biocompatibility and remarkable antitumor capabilities, making it a promising platform for localized sonopiezoelectric tumor eradication.

### 2.4 In Vitro Proliferation and Differentiation of BMSCs on the Hybrid Scaffolds

**Figure 5:**
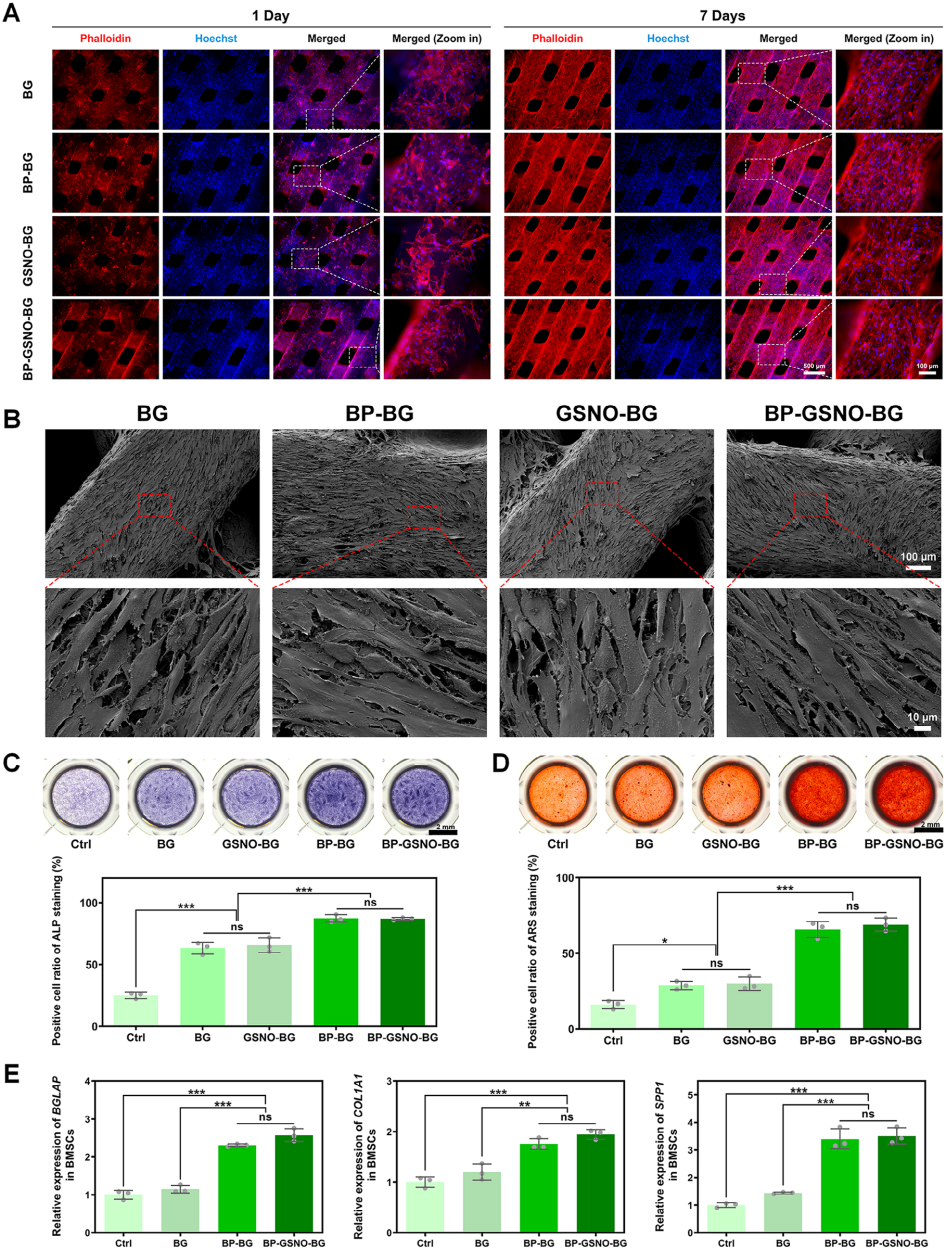
In vitro proliferation and differentiation of BMSCs on the hybrid scaffolds. A) Fluorescence images of human bone mesenchymal stem cells (BMSCs) attachment and morphology after co‐culture with the indicated scaffolds for 1 day and 7 days, respectively. BMSCs were stained with rhodamine‐phalloidin (cytoskeleton, red fluorescence) and Hoechst 33342 (cell nuclei, blue fluorescence). (Scale bar: 500 µm for low magnification fields and 100 µm for high magnification fields). B) SEM images of BMSCs cultured on the indicated scaffolds for 7 days (Scale bar: 100 µm for low magnification fields and 10 µm for high magnification fields). C) The ALP staining of BMSCs after treated with osteogenic differentiation medium for 14 days under the indicated conditions. The representative and quantitative results of staining for different groups are shown in the figure (Scale bar: 2 mm. Statistics were derived using the one‐way analysis of variance. All data were expressed as mean ± standard deviation (SD). *n* = 3 for each group, ^***^ *p* < 0.001). (D) The ARS staining of BMSCs after treated with osteogenic differentiation medium for 21 days under the indicated conditions. The representative and quantitative results of staining for different groups are shown in the figure (Scale bar: 2 mm. Statistics were derived using the one‐way analysis of variance. All data were expressed as mean ± standard deviation (SD). *n* = 3 for each group, ^*^ *p* < 0.05, ^***^ *p* < 0.001). (E) The expression levels of osteogenesis‐associated genes in BMSCs after treated with the indicated conditions for 7 days (Statistics were derived using the one‐way analysis of variance. All data were expressed as mean ± standard deviation (SD). *n* = 3 for each group, ^*^ *p* < 0.05, ^**^ *p* < 0.01, ^***^ *p* < 0.001). (Grouping for Figure C–E: Ctrl group, BMSCs cultured under normal condition; BG group, BMSCs co‐cultured with the BG scaffold; GSNO‐BG group, BMSCs co‐cultured with the GSNO‐BG scaffold; BP‐BG group, BMSCs co‐cultured with the BP‐BG scaffold; BP‐GSNO‐BG group, BMSCs co‐cultured with the BP‐GSNO‐BG scaffold).

Given the excellent biocompatibility and superior in situ biomineralization performance of BP nanosheets and bioactive glass, the osteogenic induction potential of the hybrid bioscaffolds was comprehensively investigated in vitro. The adhesion and proliferation of human bone mesenchymal stem cells (BMSCs) on hybrid bioscaffolds were assessed via fluorescent cytoskeletal staining and SEM. On day 1, a sparse layer of BMSCs scattered and adhered on the surface of the hybrid bioscaffolds, featuring well‐oriented cytoskeletal striation. This observation suggests that the unique topographical morphology and hierarchical porous architecture of BG scaffolds enhance cell attachment and proliferation (**Figure** **5**A). Furthermore, by day 7, a dense layer of BMSCs exhibiting a uniformly aligned orientation covered the surface of the hybrid scaffolds, corroborating their superior biocompatibility and desirable osteoconductivity (Figure 5A,B). Results from the CCK‐8 cell proliferation assay further confirmed that BMSCs adhered and proliferated robustly on the hybrid scaffolds during the 5‐day co‐culture period, demonstrating their excellent cytocompatibility (Figure S10, Supporting Information).

Following 14 days of co‐culture with various scaffolds, alkaline phosphatase (ALP) staining was performed on BMSCs to evaluate differences in osteogenic capability among the groups (Figure 5C). Notably, the percentage of ALP‐positive cells in the hybrid scaffold groups was significantly higher than in the scaffold‐free control group, indicating the excellent osteoinductive potential of bioactive glass scaffolds. Furthermore, the incorporation of BP nanosheets further enhanced the osteogenic differentiation of BMSCs, leading to a tremendously elevated percentage of ALP‐positive cells in BP‐BG and BP‐GSNO‐BG groups, reaching 87.32 ± 2.99% and 86.98 ± 1.03%, respectively. Alizarin red staining (ARS) was performed to visualize calcium nodule formation following 21 days of osteogenic induction. As expected, the ARS results exhibited trends similar to those observed in the ALP staining assay (Figure 5D). After 7 days of culture under various conditions, the expression levels of osteogenesis‐associated genes in BMSCs, including *BGLAP* (osteocalcin, *OCN*), *COL1A1* (collagen type I alpha 1), and *SPP1* (osteopontin, *OPN*), were analyzed via RT‐qPCR (Figure 5E). Notably, *BGLAP* expression levels in BP‐BG and BP‐GSNO‐BG groups were significantly higher than in all other groups, reaching 2.31 ± 0.03‐fold and 2.57 ± 0.17‐fold the expression level of the control group, respectively. Overall, the expression of these osteogenesis‐associated genes was markedly upregulated in the BP‐BG and BP‐GSNO‐BG groups. These findings highlight the robust osteogenic effects of BP nanosheets and bioactive glass. Collectively, the superior osteoconductivity and osteoinductivity of BP‐integrated BG scaffolds position them as promising candidates for bone regeneration, offering substantial potential in the field of regenerative medicine.

### 2.5 In Vitro Assessment of the Angiogenic Effects of Hybrid Scaffolds

Optimal bone repair requires the coupling between coordinated regeneration of both osseous tissue and vascular structures.^[^ ^21^ ^]^ Neovascularization plays a pivotal role in osteointegration, highlighting the necessity of investigating the proangiogenic potential of BP‐GSNO‐BG hybrid scaffolds. To evaluate the migration capacity of human umbilical vein endothelial cells (HUVECs), scratch wound and transwell assays were performed. Co‐culture with GSNO‐BG and BP‐GSNO‐BG scaffolds significantly enhanced HUVEC migration, suggesting that these scaffolds spontaneously release low‐concentration NO, thereby exerting a proangiogenic effect on endothelial cells (Figures S11 and S12, Supporting Information). As a paradigm, scratch wound contraction in the BP‐GSNO‐BG group reached 64.53 ± 2.72 µm within the first 6 h and 132.80 ± 1.56 µm after 24 h of co‐culture, demonstrating significantly accelerated wound closure compared to the control and BP‐BG groups (Figure S11, Supporting Information). In parallel, the tube formation capacity of HUVECs was markedly enhanced following co‐culture with GSNO‐BG and BP‐GSNO‐BG scaffolds (Figure S13, Supporting Information), in accordance with the findings from the scratch wound and transwell assays. Collectively, the robust proangiogenic effect of the BP‐GSNO‐BG hybrid scaffold may be attributed to its spontaneous release of low‐concentration NO, which effectively promotes neovascularization, supports the maturation of newly formed osseous tissue, and synergistically facilitates bone regeneration.

### 2.6 Hybrid Scaffold‐Promoted Bone Regeneration in a Cranial Defect Model

**Figure 6:**
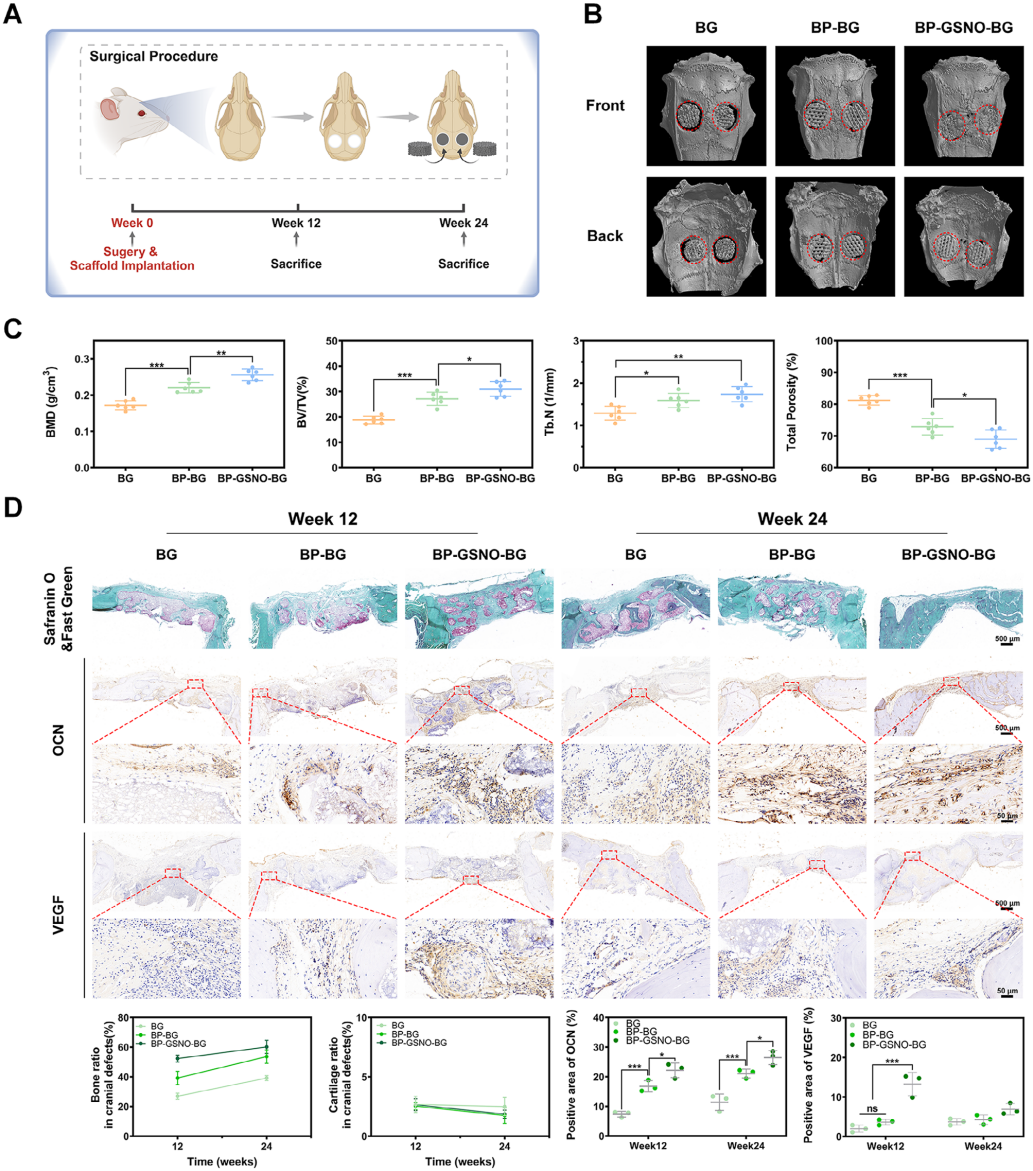
Hybrid scaffolds promote bone regeneration of cranial defects. A) Schematic illustration of rat cranial defect model. Created in BioRender. Fang, H. (2025). B) 3D reconstruction imaging of rat craniums after 24‐week implantation of hybrid scaffolds. Red circles indicate the original defected area. C) Micro‐CT analysis of cranial defected area after 24‐week implantation of hybrid scaffolds. The bone mineral density (BMD), trabecular bone volume fraction (BV/TV), trabecular number (Tb.N), and total porosity were measured from the Micro‐CT data (Statistics were derived using the one‐way analysis of variance. All data were expressed as mean ± standard deviation (SD). *n* = 6 defects for each group, ^*^ *p* < 0.05, ^**^ *p* < 0.01, ^***^ *p* < 0.001). D) Morphological staining (Safranin‐O/Fast Green) and immunohistochemical staining (OCN, VEGF) of cranium slicing from different groups. The red boxes represent the specific location of the high‐magnification fields (Scale bar: 500 µm for low‐magnification fields and 50 µm for high‐magnification fields). The quantitative results of morphological staining and immunohistochemical staining for different groups at each time point were shown in the figure (Statistics were derived using the two‐way analysis of variance. All data were expressed as mean ± standard deviation (SD). *n* = 3 for each group at each time point, ^*^ *p* < 0.05, ^***^ *p* < 0.001).

A rat cranial defect model was established to evaluate the biocompatibility, osteoconductivity, osteoinductivity, and osteointegration capacity of the hybrid scaffolds (**Figure** **6**A). Following the establishment of the cranial defect model and surgical implantation of different scaffolds, the rats were maintained and subsequently sacrificed at predefined time points (after 12 and 24 weeks of scaffold implantation) to harvest the defected craniums for radiographical, morphological, and histological assessments. The 3D reconstruction images of the harvested craniums revealed significantly greater calcified osseous tissue surrounding the defect margins, along with increased osteoid formation within the defected area in the BP‐GSNO‐BG group at week 24 (Figure 6B). To quantitatively assess bone regeneration, a variety of key parameters, including bone mineral density (BMD), bone volume to total volume ratio (BV/TV), trabecular number (Tb.N), and total porosity, were analyzed for each group (Figure 6C). Notably, the BP‐GSNO‐BG group exhibited a superior BMD (0.26 ± 0.02 g cm^−3^) along with a significantly elevated BV/TV ratio (31.02 ± 2.91%), indicating a higher percentage of newly formed osseous tissue within the defected area. Collectively, these radiographic findings suggest that BP‐GSNO‐BG scaffolds accelerate the mineralization process during intramembranous ossification and facilitate osseous tissue regeneration.

Furthermore, morphological staining and immunohistochemical analysis were performed to examine and compare specific components of the newly formed bone in different groups (Figure 6D). Safranin‐O/Fast Green staining, commonly used to distinguish cartilage (stained red) from bone (stained green), demonstrated a consistently low proportion of cartilage components in the newly formed bone across all groups throughout the regeneration process. This finding aligns with the physiological remodeling sequence of intramembranous ossification. Additionally, the proportion of trabecular and cortical bone within the defected area in the BP‐GSNO‐BG group was significantly higher than in the other groups at both week 12 and week 24, indicating a more advanced ossification process. Notably, the secretion of osteocalcin (OCN), a key marker of osteoblastic activity and an essential regulator of bone matrix mineralization,^[^ ^22^ ^]^ was markedly upregulated in the defect area of the BP‐GSNO‐BG group compared to the control group at both week 12 and week 24. This finding suggests a more active ossification process in the BP‐GSNO‐BG group (Figure 6D). Furthermore, as an indispensable proangiogenic cytokine involved in both intramembranous ossification and endochondral ossification, the expression levels of vascular endothelial growth factor (VEGF) in the defected areas of different groups were also investigated (Figure 6D). The results demonstrated that the VEGF expression level in the BP‐GSNO‐BG group was consistently higher than in the other groups throughout the regeneration process, indicating enhanced angiogenesis and ossification activity in this group. Taken together, the BP‐GSNO‐BG hybrid scaffold not only facilitates osteogenic differentiation but also promotes vascularization, thereby synergistically accelerating bone regeneration. In summary, the in situ biodegradation of BP nanosheets generates non‐toxic phosphorus‐based agents for facilitating osteogenesis and osseointegration, while the sustained release of low‐concentration NO serves as both a proangiogenic regulator and an osteogenic factor to synergistically promote bone regeneration, which establishes the BP‐GSNO‐BG hybrid scaffold as a promising platform for facilitating post‐tumor bone regeneration.

## 3 Conclusion

This study presents a hybrid 4D bioscaffold incorporating few‐layer black phosphorus (BP) and nitric oxide (NO) donors for the effective eradication of osteosarcoma and subsequent bone regeneration. This scaffold achieves a synergistic integration of BP‐based sonopiezoelectric therapy and NO‐based gas therapy, providing an ultrasound‐responsive stepwise countermeasure against tumor invasion in bone tissues. Under ultrasonic exposure, the antitumor efficacy was facilitated by ultrasound‐activated piezoelectric ROS generation from few‐layer BP nanosheets, coupled with the burst release of high‐concentration NO from GSNO. Findings from both in vitro and in vivo experiments demonstrated that ultrasound‐triggered sonopiezoelectric therapy combined with gas therapy exerted a potent synergistic effect on tumor eradication, effectively suppressing tumor growth and metastasis without inducing off‐target toxicity. Additionally, the in situ biodegradation of BP nanosheets facilitated osteogenesis and osseointegration, while the sustained release of low‐concentration NO at later stages functioned as both a proangiogenic regulator and an osteogenic factor to synergistically promote bone regeneration. Notably, this multifunctional therapeutic platform underwent dynamic self‐transformation in its functionality—shifting from antitumor activity to osteogenic stimulation—throughout the therapeutic course, exemplifying an innovative paradigm for the novel conception of 4D biomaterials.^[^ ^19b^ ^]^

In this research, we combined two distinct animal models to evaluate the therapeutic efficacy of the ultrasound‐responsive 4D bioscaffold. Subcutaneous osteosarcoma model in nude mice was established to evaluate the tumor eradication efficacy of the synergistic sonopiezoelectric‐gaseous tumor therapy, while the in vivo osteogenic capability of the multifunctional bioscaffold was investigated using the critical‐sized cranial defect model in Sprague‐Dawley rats. It is worth mentioning that an orthotopic osteosarcoma model would provide more clinical relevance and robust insights into the scaffold's efficacy by mimicking the native tumor microenvironment. However, the application of orthotopic osteosarcoma models was limited in the preclinical research due to several critical challenges, including the technical complexity of invasive tumor implantation, tumor cell leakage risks, heightened variability in tumor growth patterns, and challenges in real‐time monitoring, which might compromise experimental reproducibility and translational relevance.^[^ ^23^ ^]^ On the other hand, the heterotopic osteosarcoma model, such as the subcutaneous osteosarcoma model, was more commonly used for immunological, pharmaceutical, and therapeutic studies in the last few decades, due to its straightforward biological performance.^[^ ^23a^ ^]^ Therefore, the subcutaneous osteosarcoma model offers superior practicality for real‐time evaluation of tumor growth inhibition, which makes it a more preferable and practical choice for this multilevel research. Additionally, an ideal orthotopic osteosarcoma model should be explored in the future to simultaneously evaluate the therapeutic efficacy of multifunctional biomaterials on tumor ablation and bone reconstruction.

Despite the promising therapeutic efficacy of this ultrasound‐responsive 4D bioscaffold, several limitations and challenges must be surmounted to fully exploit its potential for clinical applications. First, histological staining of tumor sites (Figure 4D) and major organs (Figure 4E) of the mice after 14‐day scaffold implantation revealed no sign of tumor recurrence or metastasis in the BP‐GSNO‐BG + US treated group, indicating that the sustained low‐concentration NO release from BP‐GSNO‐BG scaffold would not increase the risk of residual tumor recurrence. However, the underlying off‐target effect of low‐concentration NO requires further experimental validation using the residual tumor model. Second, the tissue penetration depth of this ultrasound‐responsive multifunctional therapeutic system and its penetration capability in a bone environment should be further explored in future experiments using the deep‐seated tumor model and orthotopic osteosarcoma model. Third, the comprehensive ion release profile of the bioscaffolds should be further validated in future experiments to better comprehend the bioactive performance of the scaffolds.

Substantial efforts are still required to achieve the bench‐to‐bedside translation of this ultrasound‐responsive 4D bioscaffold. Concretely, clinical scenarios involving massive tumors, irregular tumor shapes, and tumor invasion within bone canalicular structures may emerge as daunting challenges for the translational application of this 4D bioscaffold. To optimize this ultrasound‐responsive multifunctional therapeutic system for addressing the heterogeneity of neoplastic and osseous tissues, we would like to propose a feasible design named Standardized Implant Cluster Configuration, which utilizes standardized ultrasound‐responsive 4D bioscaffolds with standardized scaffold size and quantitatively predetermined therapeutic radius. Following volumetric and morphological analysis of the tumor using imaging modalities, the scaffold implantation coordinates are computationally optimized through the corresponding algorithms. Multi‐point stereotactic scaffold implantation is then performed via minimally invasive surgeries or stereotactic injection, thereby creating an implant cluster configuration to ensure consistent tumoricidal coverage for massive and irregular‐shaped tumors. Collectively, this Standardized Implant Cluster Configuration protocol maintains excellent therapeutic predictability while allowing for patient‐specific customization. It is also worth mentioning that the ultrasound parameters (e.g., frequency, power density, and even the position and number of ultrasonic probes) could be modulated to enhance the tissue penetration depth and therapeutic efficacy of this ultrasound‐responsive multifunctional therapeutic system, enabling the precise and spatiotemporally controlled treatment for deep‐seated tumors and tumor invasion within bone canalicular structures.

In summary, the synergistic combination of BP‐based sonopiezoelectric therapy and NO‐based gas therapy proposed a novel antitumor strategy with unprecedented research value and promising clinical applications, addressing the intrinsic limitations of conventional cancer therapies and offering vast potential for the future design of antitumor strategies. Furthermore, the design of this 4D multifunctional therapeutic platform, characterized by superior sonopiezoelectric efficacy, controllable NO release, and stimulatory effects on tissue regeneration, provides new insights into the comprehensive treatment of aggressive tumors. With the continuous advancement of stimuli‐responsive multifunctional biomaterials, we anticipate that precise and spatiotemporally controlled tumor therapies with minimal side effects will emerge to address the escalating demand for effective and targeted cancer treatments, offering new hope for cancer patients worldwide.

## 4 Experimental Section

### Materials

BP crystal powder was purchased from Nanjing XFNANO Materials Tech Co., Ltd (Nanjing, China) and stored in a dark argon‐filled (Ar) glovebox to prevent oxidation. Aqueous solutions of N‐methyl‐2‐pyrrolidone (NMP), tetraethyl orthosilicate (TEOS), calcium nitrate tetrahydrate, and triethyl phosphate were obtained from Sinopharm Chemical Reagents Co., Ltd (Shanghai, China). S‐nitroso‐L‐glutathione (GSNO) was purchased from MedChemExpress LLC (Shanghai, China). 2,2,6,6‐Tetramethylpiperidine (TEMP) and 5,5‐dimethyl‐1‐pyrroline N‐oxide (DMPO) were obtained from Sigma–Aldrich (Burlington, MA, USA).

### Synthesis of BP Nanosheets

Few‐layer BP nanosheets were synthesized using a liquid‐phase exfoliation method. Specifically, 60 mg of BP crystal powder was homogenously dispersed in 160 mL of NMP using ultrasonication in an ice‐cooled bath for 24 h to prevent thermal degradation. The resulting suspension was then centrifuged at 5000 rpm for 10 min to eliminate any residual unexfoliated BP crystal powder. The supernatant, containing few‐layer BP nanosheets, was carefully collected by decantation to minimize contamination from larger unexfoliated particles.

### 3D Printing of BG Scaffolds

BG scaffolds with a uniform macroporous morphology were constructed via the 3D printing technique. First, a sol–gel precursor solution was prepared by sequentially dispersing 53.6 g of TEOS, 11.2 g of calcium nitrate tetrahydrate, 5.84 g of triethyl phosphate, and 8 g of HCl (1 m) into 480 g of absolute ethanol under continuous stirring at room temperature for 24 h. The resulting solution was then air‐dried to facilitate solvent evaporation, leading to the screening of the bioactive glass powder. Next, 1 g of bioactive glass powder was dispersed in 1 g of polyvinyl alcohol (PVA) solution (10 wt.% in H_2_O) and stirred magnetically for 30 min to obtain a homogeneous printing mixture. The prepared mixture was then loaded into a printing tube and 3D‐printed using a precision printing needle to fabricate the raw bioactive glass (BG) scaffolds. Finally, the printed scaffolds underwent sintering at 1060 °C for 12 h to achieve structural integrity and enhance bioactivity.

### Preparation of Hybrid Scaffolds

To fabricate BP‐BG hybrid scaffolds, the sintered BG scaffolds were soaked in the BP absolute ethyl alcohol solution for 12 h, followed by air‐drying at room temperature. This process was repeated three times to ensure uniform BP deposition and obtain the final BP‐BG hybrid scaffolds. Similarly, to fabricate GSNO‐BG hybrid scaffolds, the sintered BG scaffolds underwent a soaking process in the GSNO dispersion solution for 6 h, followed by air‐drying at room temperature. The aforementioned procedure was also repeated three times to ensure adequate incorporation of GSNO. For the fabrication of BP‐GSNO‐BG hybrid scaffolds, the previously obtained BP‐BG hybrid scaffolds were further immersed in the GSNO dispersion solution for 6 h, followed by air‐drying at room temperature. The aforementioned procedure was also repeated three times to achieve uniform surface functionalization and obtain the final BP‐GSNO‐BG hybrid scaffolds.

### Characterization of Few‐Layer BP Nanosheets and Hybrid Scaffolds

The transmission electron microscopy (TEM) images and high‐resolution scanning transmission electron microscopy (STEM) images of BP nanosheets were captured using a JEM‐ARM200F atomic resolution analytical electron microscope (JEOL Ltd., Tokyo, Japan). Piezoresponse force microscopy (PFM) characterization of BP nanosheets was carried out by adding few‐layer BP nanosheet dispersion (0.1 mg mL^−1^ in Millipore water) in drops onto a conducting substrate, followed by air‐drying at room temperature and then measuring PFM characteristics using a Dimension Icon piezoresponse force microscope (Bruker, Billerica, MA, USA). UV–vis–NIR diffuse reflectance spectroscopy of few‐layer BP nanosheets was conducted on a UV‐3600i Plus UV–vis–NIR spectrophotometer (Shimadzu, Kyoto, Japan). The scanning electron microscopy (SEM) images and corresponding elemental mapping analysis of the hybrid scaffolds were performed using an SU8220 field‐emission scanning electron microscope (Hitachi, Tokyo, Japan).

### Electron Spin Resonance (ESR) Spectroscopy of Few‐Layer BP Nanosheets

To evaluate the ROS‐generating capability of BP nanosheet dispersion under ultrasonic stimulation, electron spin resonance (ESR) spectroscopy was performed. For the detection of hydroxyl radicals (•OH), few‐layer BP nanosheet dispersion (50 µg mL^−1^ in PBS, 1 mL) was mixed with 10 µL of DMPO and then exposed to the ultrasonic stimulation (1 MHz, 1.2 W cm^−2^) for 60 s. For the detection of singlet oxygen (^1^O_2_), few‐layer BP nanosheet dispersion (50 µg mL^−1^ in PBS, 1 mL) was mixed with 20 µL of TEMP and then exposed to the ultrasonic stimulation (1 MHz, 1.2 W cm^−2^) for 60 s. To detect superoxide radical anion (O_2_^•−^), few‐layer BP nanosheet dispersion (50 µg mL^−1^ in methanol, 1 mL) was mixed with 20 µL of DMPO and exposed to the ultrasonic stimulation (1 MHz, 1.2 W cm^−2^) for 60 s. The characteristic ESR peak signals of each ROS species were recorded using an EMXplus ESR spectrometer (Bruker, Billerica, MA, USA).

### Cell Culture and In Vitro Cytotoxicity Assay

MG63 osteosarcoma cells, human umbilical vein endothelial cells (HUVECs), and human bone marrow mesenchymal stem cells (BMSCs) were obtained from the Cell Bank of the Chinese Academy of Sciences (Shanghai, China). MG63 cells were cultured in Dulbecco's Modified Eagle Medium (DMEM) (Gibco BRL, Grand Island, NY, USA) supplemented with 10% fetal bovine serum (FBS) (Gibco BRL) and 100 mg mL^−1^ penicillin and streptomycin (Gibco BRL). BMSCs were cultured in Minimum Essential Medium Alpha (α‐MEM) (Gibco BRL, Grand Island, NY, USA) supplemented with 10% FBS (Gibco BRL) and 100 mg mL^−1^ penicillin and streptomycin (Gibco BRL). Cells were cultured in an incubator at 37 °C with a humidified atmosphere of 5% CO_2_. Cell proliferation was assessed using the Cell Counting Kit‐8 (CCK‐8) (Dojindo, Kumamoto, Japan). Cells were seeded at a density of 5 × 10^3^ well^−1^ in a 96‐well plate. After co‐culturing with different groups of scaffolds respectively for predetermined time intervals, CCK‐8 solution was added to the plates and incubated for 2 h. The absorbance of each well was measured using Varioskan LUX (Thermo Fisher Scientific, Waltham, MA, USA). Live/dead cell staining assay was performed to assess cell viability using the Calcein/PI Live/Dead Viability/Cytotoxicity Assay Kit (Beyotime, Shanghai, China). Live cells were stained with Calcein‐AM (green fluorescence), and dead cells were stained with propidium iodide (PI) (red fluorescence). The stained samples were observed under a fluorescence microscope.

### In Vitro Sonodynamic Ablation of Osteosarcoma Cells

MG63 cells grown on BG scaffolds, BP‐BG scaffolds, and BP‐GSNO‐BG scaffolds were cultured under indicated conditions with/without ultrasonic stimulation. To evaluate the efficacy of sonodynamic therapy (SDT) in vitro, CCK‐8 assays were performed on the MG63 cells after different treatments, as mentioned above. Various SDT parameters, including irradiation duration (0, 30, 60, 90, 120 s) and power density (0, 0.72, 0.96, 1.2 W cm^−2^), were systematically evaluated. For live/dead viability/cytotoxicity fluorescence imaging, the different scaffolds were placed in 48‐well plates, and MG63 cells were gently seeded on different bioscaffolds at a density of 5.0 × 10^4^ cells per scaffold. Following different treatments, the medium was carefully removed, and the cell‐laden scaffolds were gently rinsed with phosphate‐buffered saline (PBS) before being fixed with 4% paraformaldehyde for 30 min. The fixed cell‐laden scaffolds were then stained with Calcein‐AM (live cells) and propidium iodide (PI) (dead cells), and fluorescence images were captured using a DMi8 inverted microscope (Leica, Wetzlar, Germany). ROS generation was detected using the DCFH‐DA Reactive Oxygen Species Assay Kit (Beyotime, Shanghai, China) following the manufacturer's instructions. NO generation was detected using the DAF‐FM‐DA fluorescence probe (Beyotime, Shanghai, China), following the manufacturer's instructions.

### High‐Throughput RNA Sequencing

After the indicated treatment, MG63 cells were harvested for mRNA extraction and quantification via high‐throughput sequencing. Total RNA of MG63 cells was carefully extracted using the RNAqueous‐Midi Kit (AM1911, Thermo Fisher Scientific, Waltham, MA, USA) following the manufacturer's instructions. After RNA purification, high‐throughput sequencing was performed in triplicates via the Illumina HiSeq X Ten Second Generation Sequencing Platform (Seq‐Health Co., Wuhan, China). (Treatment group: MG63 cells co‐cultured with the BP‐GSNO‐BG hybrid scaffold for 24 h and subjected to ultrasound stimulation (1 MHz, 1.2 W cm^−2^) for 30 s. Control (Ctrl) group: MG63 cells were cultured under normal conditions and subjected to the same ultrasound stimulation (1 MHz, 1.2 W cm^−2^) for 30 s. Three biological replicates were collected for each group.)

### Establishment of a Subcutaneous Osteosarcoma Model

Female BALB/c nude mice (6 weeks old, ≈18 g in weight) were used for the establishment of subcutaneous osteosarcoma model. Care and use of laboratory animals were approved by the Animal Research Committee of Shanghai Sixth People's Hospital Affiliated to Shanghai Jiao Tong University School of Medicine (Approval No. 2023‐0204). MG63 cells (5 × 10^6^ cells per site) were suspended in PBS and subcutaneously injected into the axillary region of mice to introduce subcutaneous osteosarcoma. Once the tumor volume reached ≈200 mm^3^, the mice were randomly divided into six groups (*n* = 5): 1) BG group, 2) BG + US group, 3) BP‐BG group, 4) BP‐BG + US group, 5) BP‐GSNO‐BG group, and 6) BP‐GSNO‐BG + US group. After anesthetizing the mice with isoflurane inhalation, the area near the axilla was disinfected. A skin incision was made by the edge of the subcutaneous tumor, followed by the implantation of different bioscaffolds (Φ5 mm × 1.5 mm) into the tumor site. The wound was carefully sutured, and intraperitoneal antibiotic administration was performed post‐surgery. For mice in the BG + US, BP‐BG + US, and BP‐GSNO‐BG + US groups, ultrasound stimulation (1 MHz, 1.2 W cm^−2^ for 10 min) was applied on Day 0, Day 1, and Day 2 after scaffold implantation. The tumor volume was continuously monitored every two days for the following 14 days using a digital caliper. Tumor volume was calculated according to the formula: Tumor volume = (tumor length) × (tumor width)^2^/2 – scaffold volume. After 14 days of treatment, the tumors were harvested, fixed with 4% paraformaldehyde for 24 h, and then sectioned into slices. H&E staining and Ki67 staining were performed for histological analysis. To further evaluate the in vivo toxicity of the hybrid scaffolds, the major organs of mice, including the lung, liver, spleen, kidney, and intestine, were dissected, fixed in 4% paraformaldehyde, and then sectioned into slices for H&E staining and histological analysis.

### Quantitative Real‐Time Polymerase Chain Reaction (RT‐qPCR)

Total mRNA was carefully extracted from BMSCs using the EZBioscience EZ‐press RNA Purification Kit B0004DP (EZBioscience, Roseville, MN, USA) following the provided instructions. The RT‐qPCR reaction mixture was prepared using the EZBioscience 2 × Color SYBR Green qPCR Master Mix (ROX2 plus) A0012‐R2 (EZBioscience, Roseville, MN, USA). In brief, the reaction mixture contained 5 µL of Color SYBR Green qPCR Master Mix, 4 µL of cDNA template, 0.2 µL of upstream primers, 0.2 µL of downstream primers, and 0.6 µL of double‐distilled water (ddH_2_O). Subsequently, RT‐qPCR was conducted on an ABI 7900 HT Sequence Detection System (Thermo Fisher Scientific, Waltham, MA, USA).

### Characterization of BMSC Adherence on the Hybrid Scaffolds

To investigate the morphology and adhesion of BMSCs on the hybrid scaffolds, different scaffolds were placed in 48‐well plates and BMSCs were gently seeded onto them at a density of 5.0 × 10^4^ cells per scaffold. After co‐culture for 1 day and 7 days, the medium was carefully removed and the cell‐laden bioscaffolds were gently rinsed with PBS, followed by fixation with 4% paraformaldehyde for 30 min. To further examine cytoskeletal morphology, the fixed cell‐laden bioscaffolds were stained with rhodamine‐phalloidin (for actin filaments) and Hoechst 33342 (for nuclei), and the fluorescence images were captured via the DMi8 inverted microscope (Leica, Wetzlar, Germany).

Furthermore, the unique topographical morphology of the cell‐laden scaffolds was investigated using SEM imaging. After co‐culture on different hybrid scaffolds for 7 days, BMSCs were then fixed with 2.5% glutaraldehyde at 4 °C for 12 h. The fixed cell‐laden bioscaffolds were then gently rinsed three times with PBS, followed by post‐fixation in 1% osmium tetroxide (OsO_4_) solution for 1 h. Subsequently, the samples were gently rinsed again with PBS and dehydrated with gradient concentrations of ethanol series (30%, 50%, 70%, 80%, 90%, 95%, and 100% v/v), followed by the dry‐out process in a Quorum K850 critical point dryer (Quorum, East Sussex, UK). Next, the dried samples were sputtered with platinum using the MC1000 ion sputter coater (Hitachi, Tokyo, Japan) and observed under a Regulus8100 scanning electron microscope (Hitachi, Tokyo, Japan).

### Osteogenic Differentiation, Alkaline Phosphatase (ALP) Staining, and Alizarin Red (ARS) Staining

The osteogenic differentiation medium was prepared by supplementing the BMSC culture medium with 10 nmol L^−1^ of dexamethasone (Sigma–Aldrich, Burlington, MA, USA), 10 mmol L^−1^ of β‐glycerophosphate (Sigma–Aldrich), and 50 µg mL^−1^ of ascorbic acid (Sigma–Aldrich). BMSCs were evenly seeded in a 96‐well plate and cultured with the osteogenic differentiation medium. After co‐culture with different bioscaffolds for 14 days, BMSCs were gently rinsed three times with PBS, fixed with 4% paraformaldehyde, and then stained with the BCIP/NBT alkaline phosphatase color development kit (Beyotime, Shanghai, China) following the manufacturer's instructions. After co‐culture with different scaffolds for 21 days, BMSCs were gently rinsed three times with PBS, fixed with 4% paraformaldehyde, and then stained with 2% Alizarin Red S staining solution (Beyotime, Shanghai, China) following the manufacturer's instructions. Images were acquired using a light microscope (Leica SAPO, Wetzlar, Germany) equipped with the Leica MC190 HD imaging system (Leica, Wetzlar, Germany). Quantitative analysis was performed using Image‐Pro Plus software.

### In Vitro Vascularization Assay

For the scratch wound assay, HUVECs were cultured in a six‐well plate until they reached confluence. A 200 µL sterile pipette tip was used to create a cell‐free area in the center of each well. The plate was gently rinsed three times with PBS to remove the cellular debris. Thereafter, HUVECs were cultured in basal medium containing 1% FBS and co‐cultured with different scaffolds as indicated. The scratch wound images were captured at predetermined time points using a Nikon Eclipse Ts2R microscope (Nikon, Tokyo, Japan) equipped with the Nikon Ds‐Ri2 imaging system (Nikon, Tokyo, Japan).

For the evaluation of cell migration in the transwell assay, HUVECs (3 × 10^4^ well^−1^) were suspended in 200 µL basal medium containing 1% FBS. The cells were then evenly seeded into the upper chambers of a transwell plate (Corning, China), followed by the placement of different bioscaffolds in the lower chambers with basal medium containing 5% FBS. After the 24 h incubation, the remaining cells in the upper chambers were fixed with 4% paraformaldehyde for 30 min, gently rinsed with PBS three times, and then stained with 0.5% crystal violet (Beyotime, Shanghai, China) for 2 min. Images of cell migration in the transwell assay were captured using a Nikon Eclipse Ts2R microscope (Nikon, Tokyo, Japan) equipped with the Nikon Ds‐Ri2 imaging system (Nikon, Tokyo, Japan).

For the tube formation assay, HUVECs were evenly seeded in a 96‐well plate, which was pre‐coated with cold extracellular matrix (ECM) gel (Sigma–Aldrich). Thereafter, the HUVECs were cultured with basal medium containing 1% FBS, followed by incubation with the leaching solution from different scaffolds for 2 h. The formation of capillary‐like tube structures was evaluated by acquiring images using a Nikon Eclipse Ts2R microscope (Nikon, Tokyo, Japan) equipped with the Nikon Ds‐Ri2 imaging system (Nikon, Tokyo, Japan).

### Modeling of Cranial Defect

A total of eighteen male Sprague‐Dawley rats (8 weeks old, ≈250 g in weight) were used to establish the cranial defect model. Care and use of laboratory animals were approved by the Animal Research Committee of Shanghai Sixth People's Hospital Affiliated to Shanghai Jiao Tong University School of Medicine (Approval No. 2023‐0204). The rats were randomly divided into three groups: 1) BG group, 2) BP‐BG group, and 3) BP‐GSNO‐BG group. After anesthetizing the rats with isoflurane inhalation, the area near the cranium was shaved and disinfected. A small incision of ≈1 cm long was made above the cranium. Two 5‐mm cranial defects were created with an electric trephine (Nouvag AG, Goldach, Switzerland), and the designated scaffolds were implanted into the defects, respectively. The wound was carefully sutured, and antibiotics were administered intraperitoneally post‐surgery. No infections or other complications were observed throughout the experiment. All mice were included in the data analysis.

### Micro‐Computed Tomography (micro‐CT) Imaging

The harvested craniums were fixed in the 4% paraformaldehyde solution overnight. A Micro‐CT scan was performed using the Skyscan 1176 micro‐CT scanner (Bruker‐microCT, Kontich, Belgium) at a voxel size of 9 µm. The acquired data were then reconstructed using CTvox and Mimics software. The bone mineral density (BMD), bone volume/total volume (BV/TV), total porosity, and trabecular number (Tb.N) were quantified using DataViewer and CTan software (Thresholding value: 60).

### Histologic and Immunohistochemical Analyses

After micro‐CT scanning, the harvested craniums were decalcified with 10% ethylenediaminetetraacetic acid solution for a duration of 4 weeks. Subsequently, the specimens were embedded in paraffin and sectioned into 5‐µm‐thick slices. For morphological assessment, Safranin‐O/Fast Green staining was performed to evaluate the cartilage and bone matrix composition. Images were acquired using a Leica DM4000 microscope (Leica, Wetzlar, Germany).

Immunohistochemical staining was performed on the slices to evaluate the expression levels of osteocalcin (OCN) and vascular endothelial growth factor (VEGF). Antibodies targeting OCN and VEGF were procured from Abcam (Cambridge, UK). The images were captured using a 3DHISTECH Pannoramic MIDI system (3DHISTECH Ltd., Budapest, Hungary).

### Statistical Analysis

Statistical analyses were conducted using GraphPad Prism 9 and Stata 15 software. All data were expressed as mean ± standard deviation (SD) from at least three independent experiments. The Unpaired Student's t‐test was utilized to determine the significant difference between the two groups, while analysis of variance (ANOVA) was conducted for multiple group comparisons. The assumptions of normality and homogeneity of variance were verified for all t‐tests and ANOVAs. Shapiro‐Wilk test was used for verifying normality and Bartlett's test was used for verifying homogeneity of variance. A *p‐*value of ≥ 0.05 indicated no statistical significance, denoted as “ns”. A *p*‐value of < 0.05 indicated statistical significance, denoted as “^*^”. A *p*‐value of < 0.01 indicated a moderately significant statistical difference, denoted as “^**^”. A *p*‐value of < 0.001 indicated a highly significant statistical difference, denoted as “^***^”.

### Ethics Approval

All procedures for the care and use of laboratory animals were approved by the Animal Research Committee of Shanghai Sixth People's Hospital Affiliated to Shanghai Jiao Tong University School of Medicine (Approval No. 2023‐0204). All animal housing and experiments were conducted in strict accordance with the institutional Guidelines for Care and Use of Laboratory Animals at Shanghai Sixth People's Hospital Affiliated to Shanghai Jiao Tong University School of Medicine.

## Conflict of Interest

The authors declare no conflict of interest.

## Author Contributions

H.F., D.Z., and Y. C. are co‐first authors and contributed equally to this work. H.F. performed conceptualization, investigation, methodology, data curation, formal analysis, visualization, funding acquisition, writing–original draft, writing–review, and editing. D.Z. performed investigation, methodology, formal analysis, visualization, writing–original draft, writing–review, and editing. Yixuan C. performed methodology, data curation, visualization, writing–review, and editing. C.Z. performed methodology, validation, writing–review, and editing. G.L. performed investigation, methodology, writing–review, and editing. Q.F. performed investigation, methodology, writing–review, and editing. M.C. performed conceptualization, investigation, validation, project administration, writing–review, and editing. Yu C. performed conceptualization, methodology, validation, project administration, supervision, writing–review, and editing. Y.G. performed conceptualization, project administration, supervision, validation, funding acquisition, writing–review, and editing.

## Supporting information



Supporting Information

## Data Availability

The data that support the findings of this study are available from the corresponding author upon reasonable request.
