# Supplementary material for: Ultrasound‐Responsive 4D Bioscaffold for Synergistic Sonopiezoelectric‐Gaseous Osteosarcoma Therapy and Enhanced Bone Regeneration
Source: Adv Sci (Weinh). 2025 Apr 3;12(22):2417208. doi: 10.1002/advs.202417208 (PMC12165076; doi:10.1002/advs.202417208)
Supplement: Supplementary file 1 — Supporting Information [file ADVS-12-2417208-s001.docx]

Supporting Information

**Ultrasound-Responsive 4D Bioscaffold for Synergistic Sonopiezoelectric-Gaseous Osteosarcoma Therapy and Enhanced Bone Regeneration**

*Haoyu Fang, Daoyu Zhu, Yixuan Chen, Changqing Zhang, Gan Li, Qihang Fang, Meiqi Chang,* Yu Chen,* and Youshui Gao**

H. Fang, D. Zhu, Yixuan Chen, C. Zhang, G. Li, Q. Fang, Y. Gao

Department of Orthopedic Surgery, Shanghai Sixth People's Hospital Affiliated to Shanghai Jiao Tong University School of Medicine, Shanghai 200233, China

Email: [gaoyoushui@sjtu.edu.cn](mailto:gaoyoushui@sjtu.edu.cn) (Y. Gao*)

M. Chang

Laboratory Center, Shanghai Municipal Hospital of Traditional Chinese Medicine, Shanghai University of Traditional Chinese Medicine, Shanghai 200071, China

Email: [changmeiqi@vip.sina.com](mailto:changmeiqi@vip.sina.com) (M. Chang*)

Yu Chen

Materdicine Lab, School of Life Sciences, Shanghai University, Shanghai 200444, China

Email: [chenyuedu@shu.edu.cn](mailto:chenyuedu@shu.edu.cn) (Yu Chen*)

**Supplementary Figures**


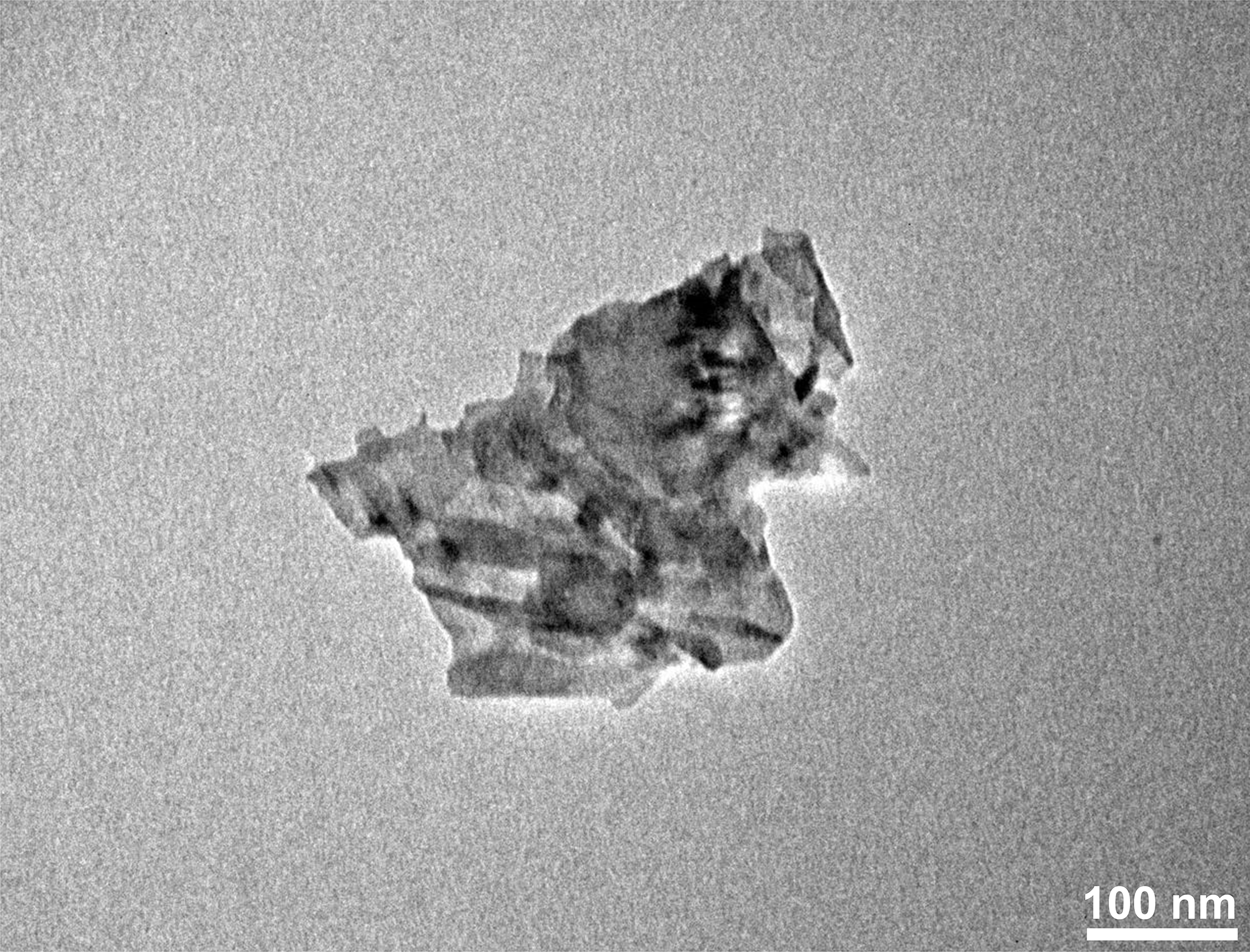


**Figure S1.** Transmission electron microscopy (TEM) image of BP nanosheets.


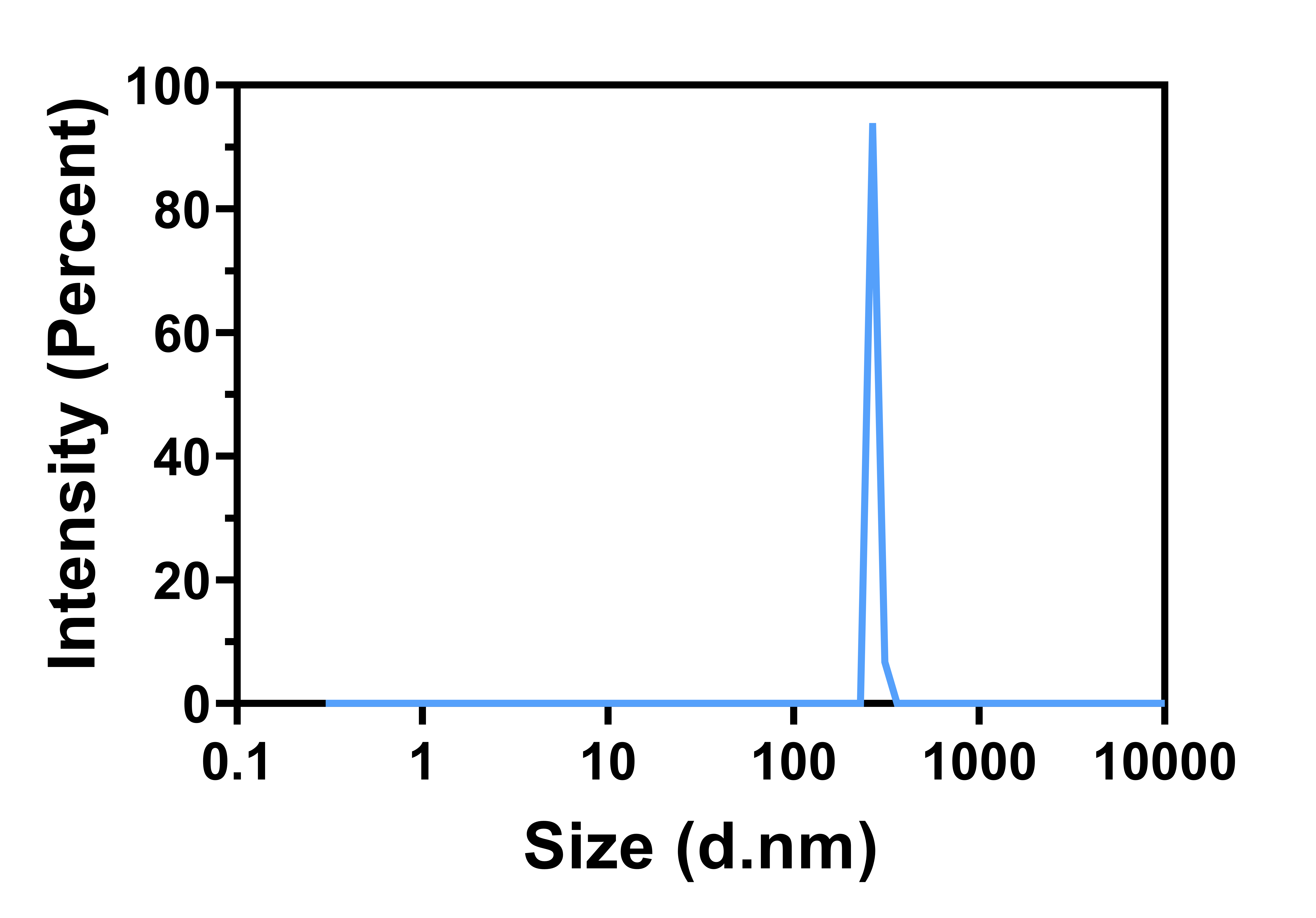


**Figure S2.** Lateral size distribution of the as-prepared BP nanosheets.


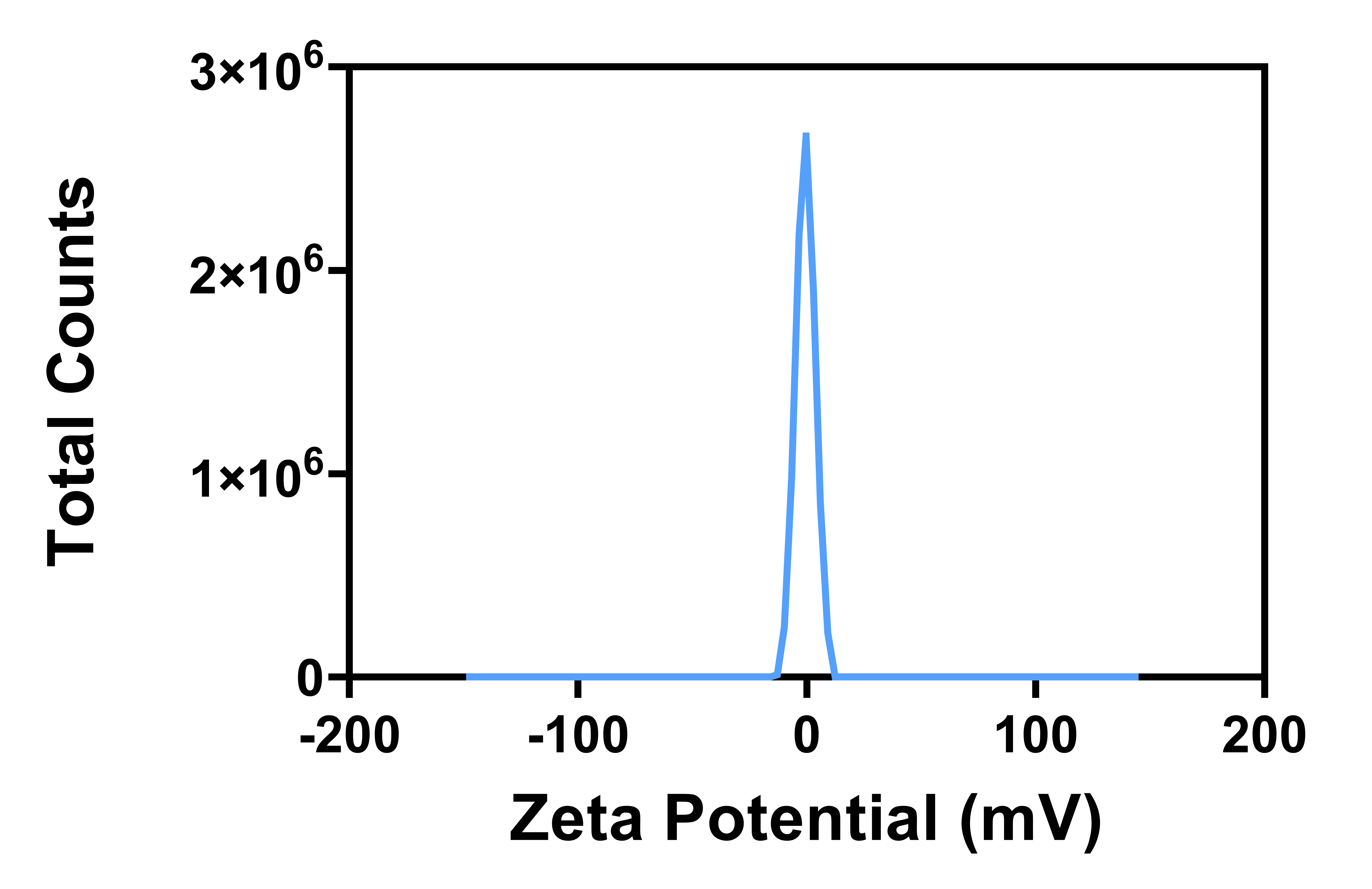


**Figure S3.** Zeta potential analysis of the as-prepared BP nanosheets.


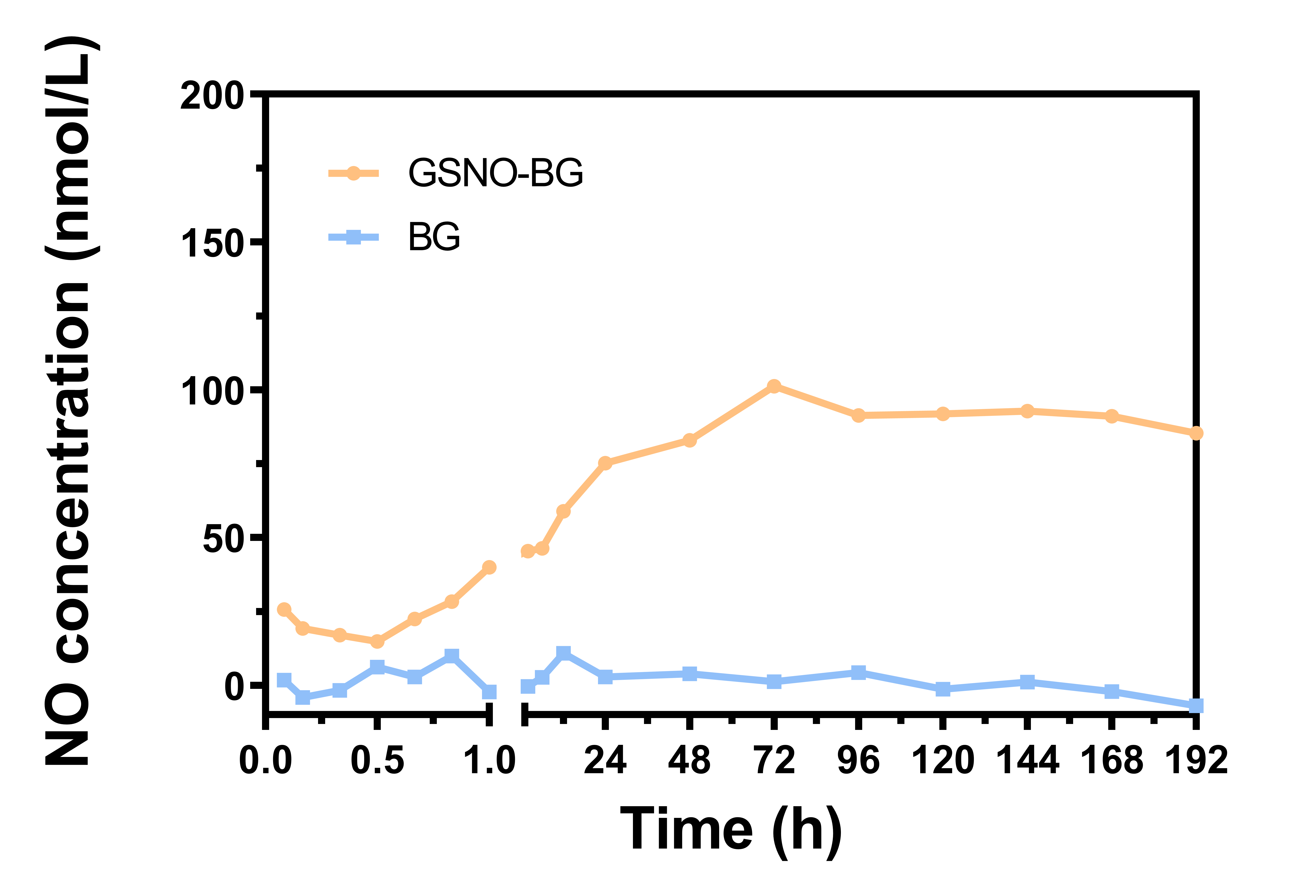


**Figure S4.** NO release efficiency of the GSNO-BG hybrid scaffold without ultrasonic stimulation.


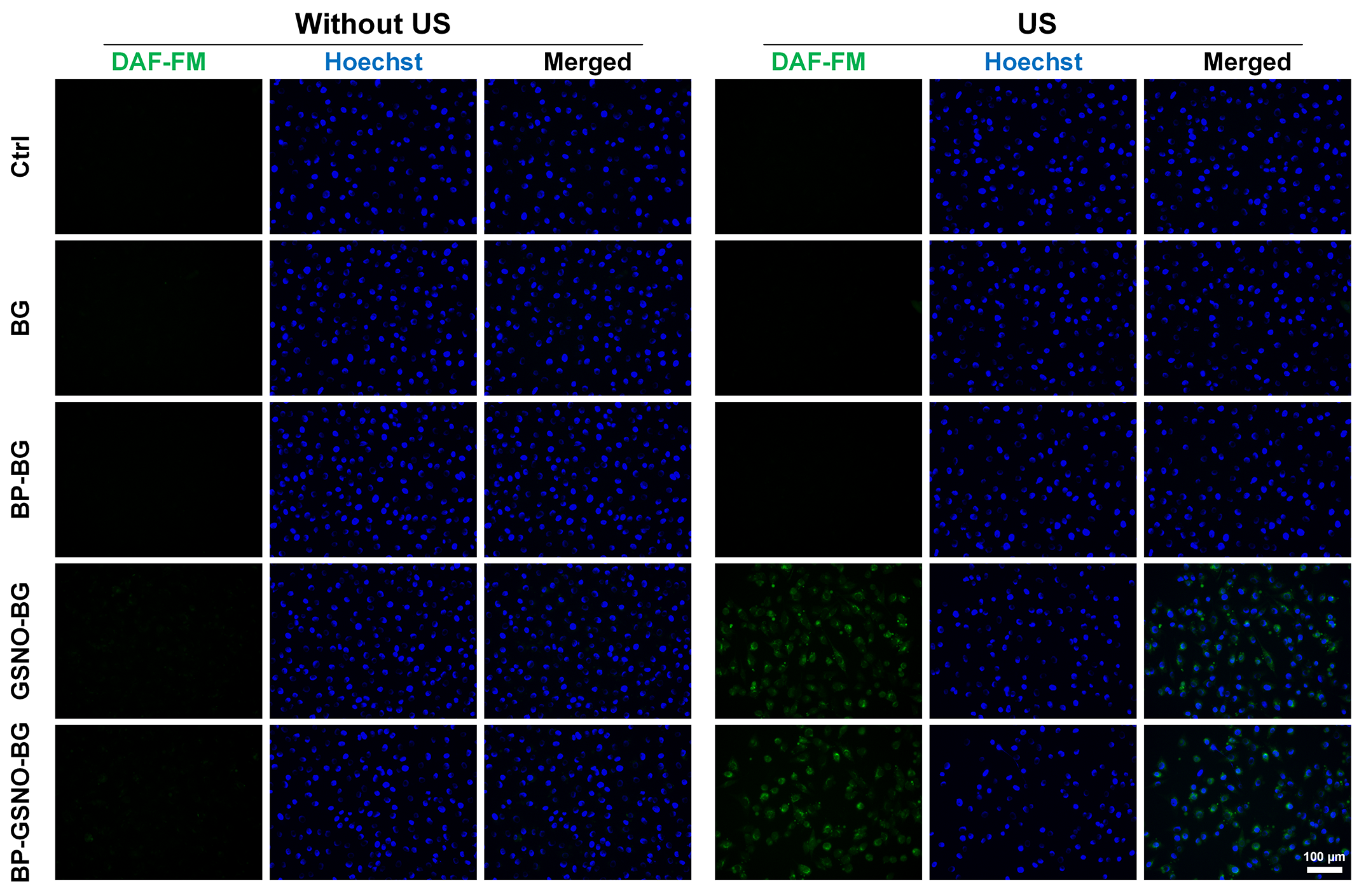


**Figure S5.** Representative NO staining (green fluorescence-DAF-FM probe) of MG63 cells treated with different groups (Scale bar: 100 μm. US refers to ultrasound stimulation (1 MHz) at 1.2 W/cm^2^ for 30 s).


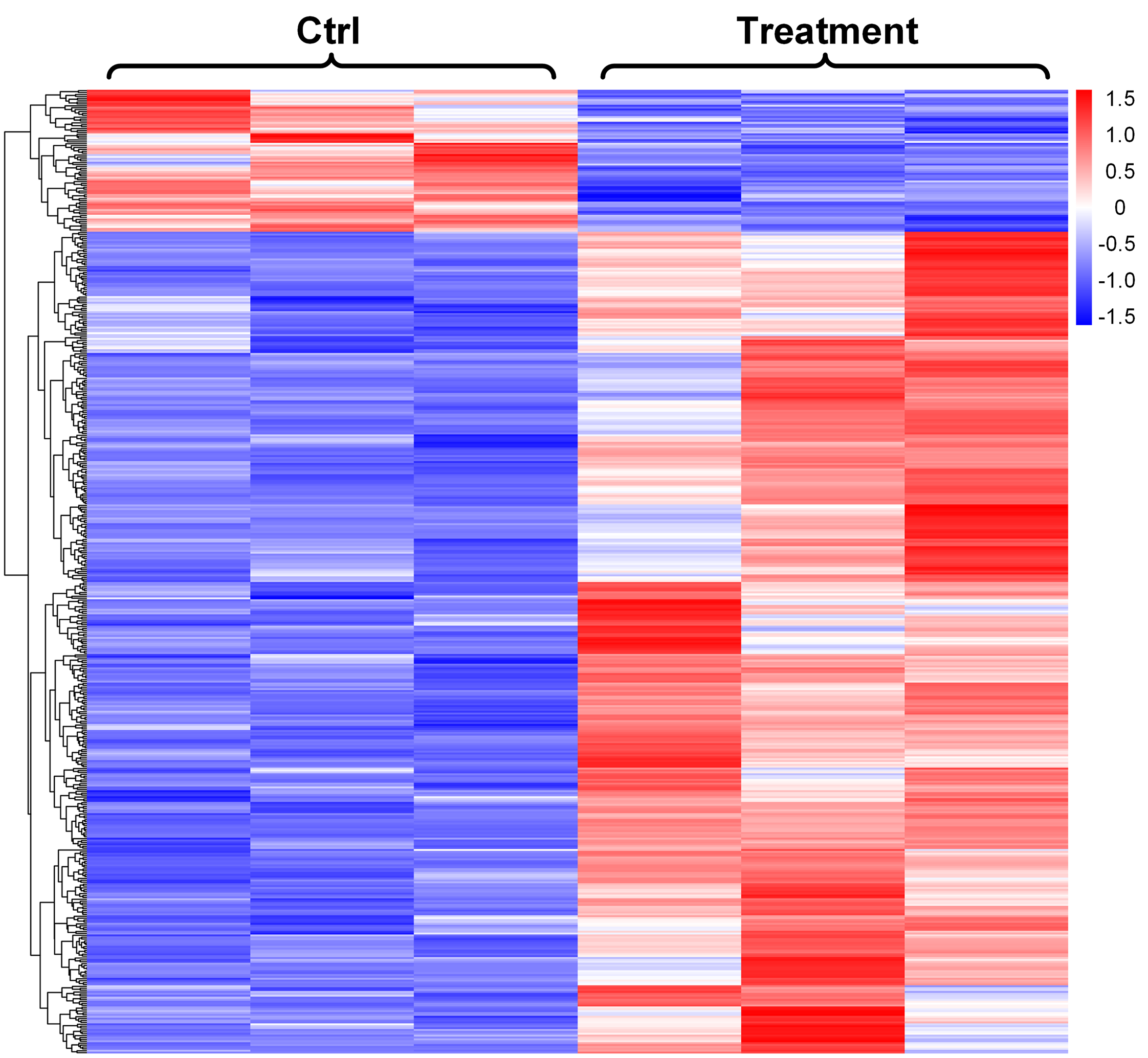


**Figure S6.** Heatmap of differential gene from the high throughput analyses.


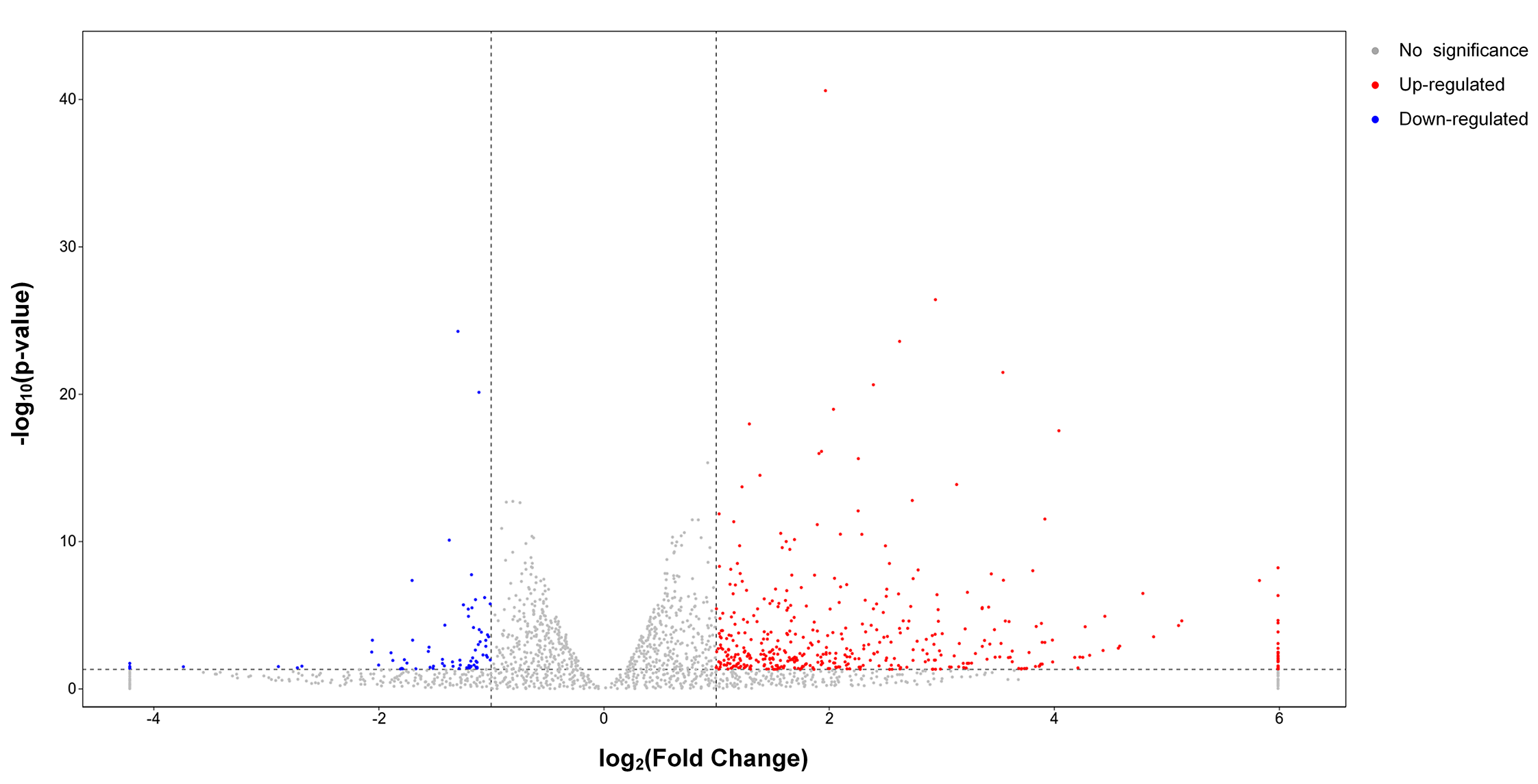


**Figure S7.** Volcano map of differential gene from the high throughput analyses.


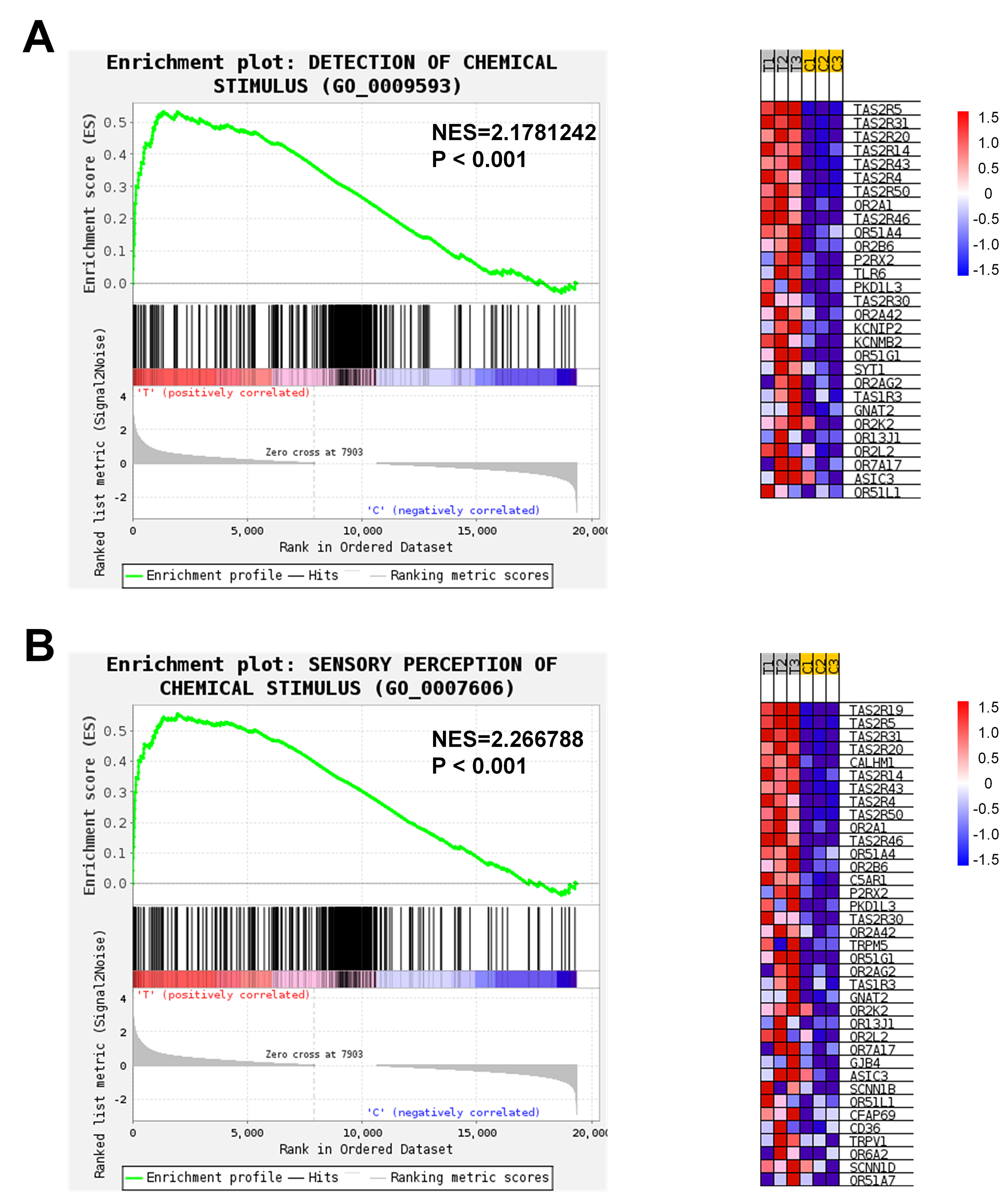


**Figure S8.** Gene set enrichment analysis (GSEA) of various pathways. (A) GSEA results of “Detection of chemical stimulus” pathway and the related genes. (B) GSEA results of “Sensory perception of chemical stimulus” pathway and the related genes.


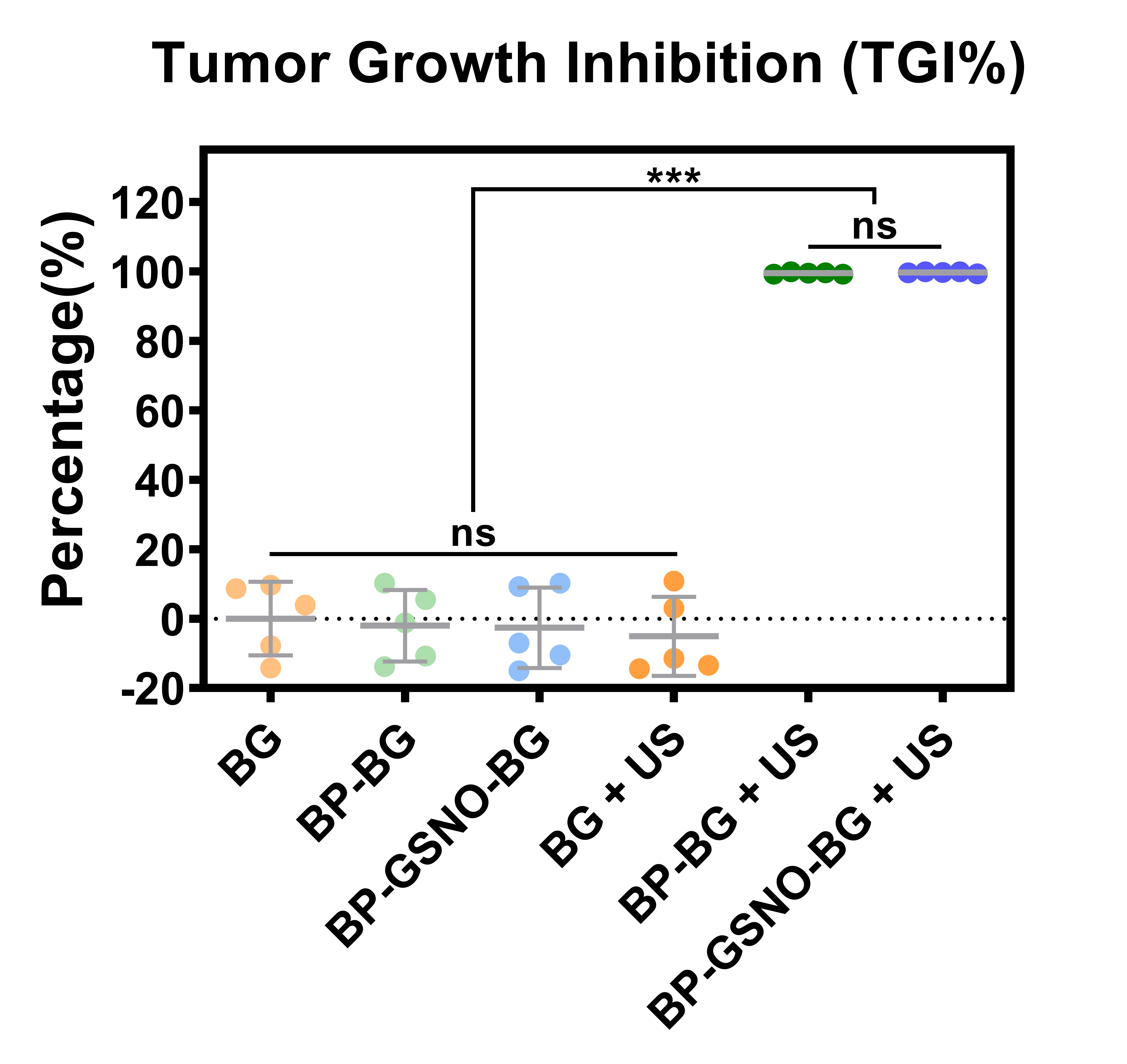


**Figure S9.** Tumor growth inhibition values of different groups after indicated treatments (Statistics were derived using the one-way analysis of variance. All data were expressed as mean ± standard deviation (SD). n = 5 for each group, ***P < 0.001).


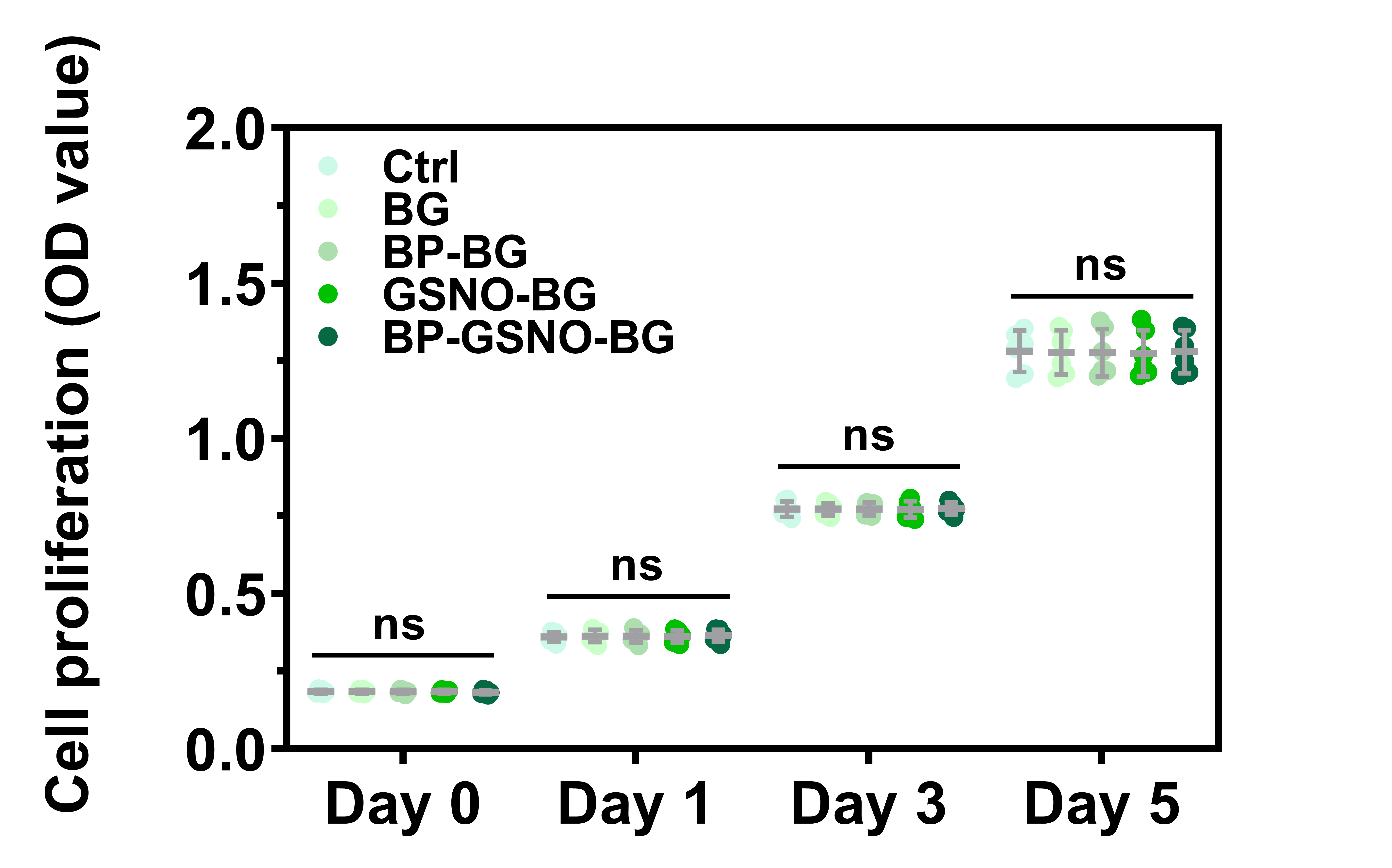


**Figure S10.** CCK-8 cell proliferation assay of BMSCs under different culture conditions (Statistics were derived using the two-way analysis of variance. All data were expressed as mean ± standard deviation (SD). n=6 for each group. ns, no significance).


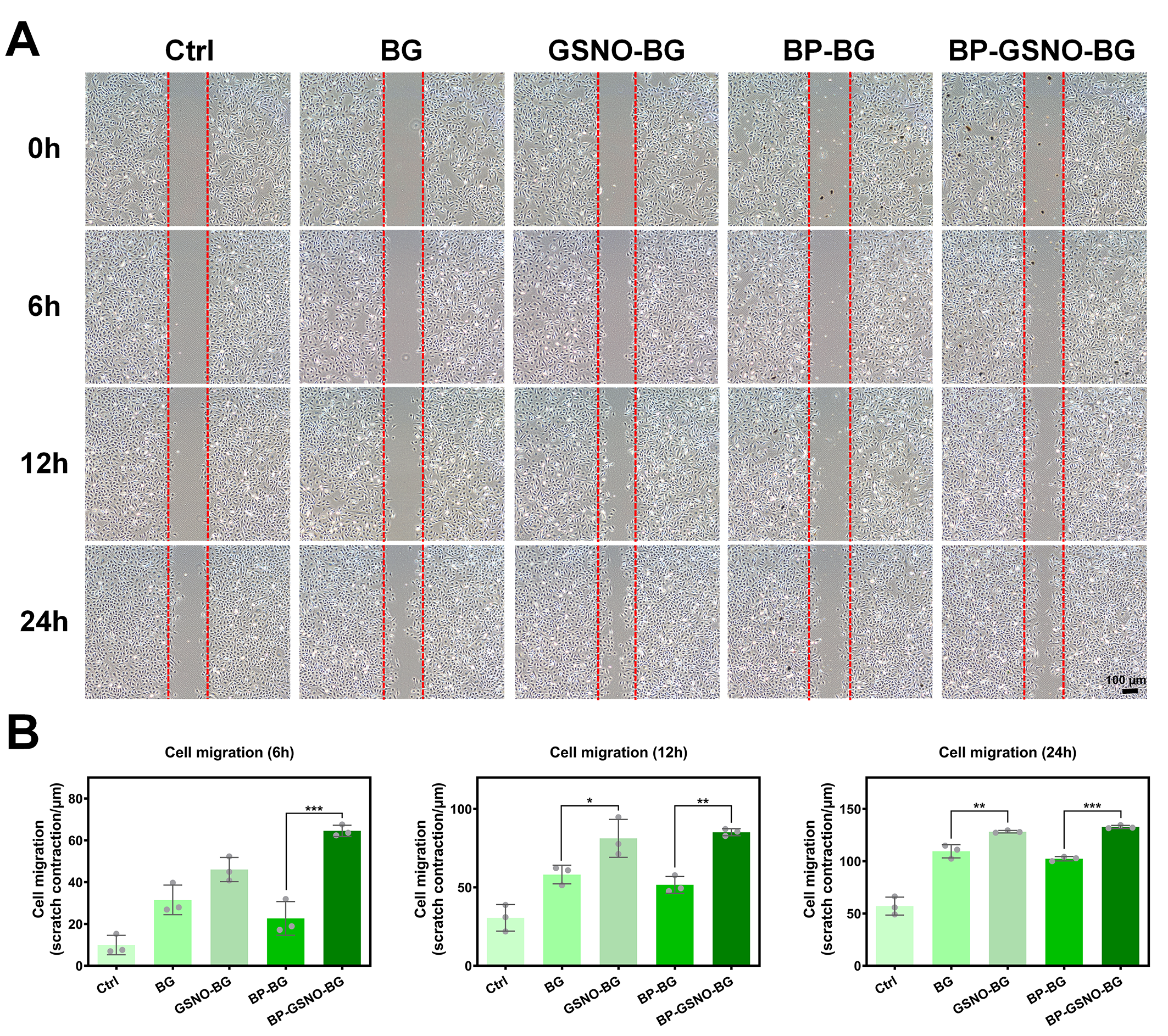


**Figure S11.** *In vitro* assessment of angiogenic effects of the hybrid scaffolds. (A) Scratch wound assay for measuring the migration capability of human umbilical vein endothelial cells (HUVECs). (Scale bar: 100 µm. Grouping: Ctrl group, HUVECs cultured under normal condition; BG group, HUVECs co-cultured with the BG scaffold; GSNO-BG group, HUVECs co-cultured with the GSNO-BG scaffold; BP-BG group, HUVECs co-cultured with the BP-BG scaffold; BP-GSNO-BG group, HUVECs co-cultured with the BP-GSNO-BG scaffold). (B) Quantification results of the scratch wound assay (Statistics were derived using the one-way analysis of variance. All data were expressed as mean ± standard deviation (SD). n = 3 for each group, *P < 0.05, **P < 0.01, ***P < 0.001).


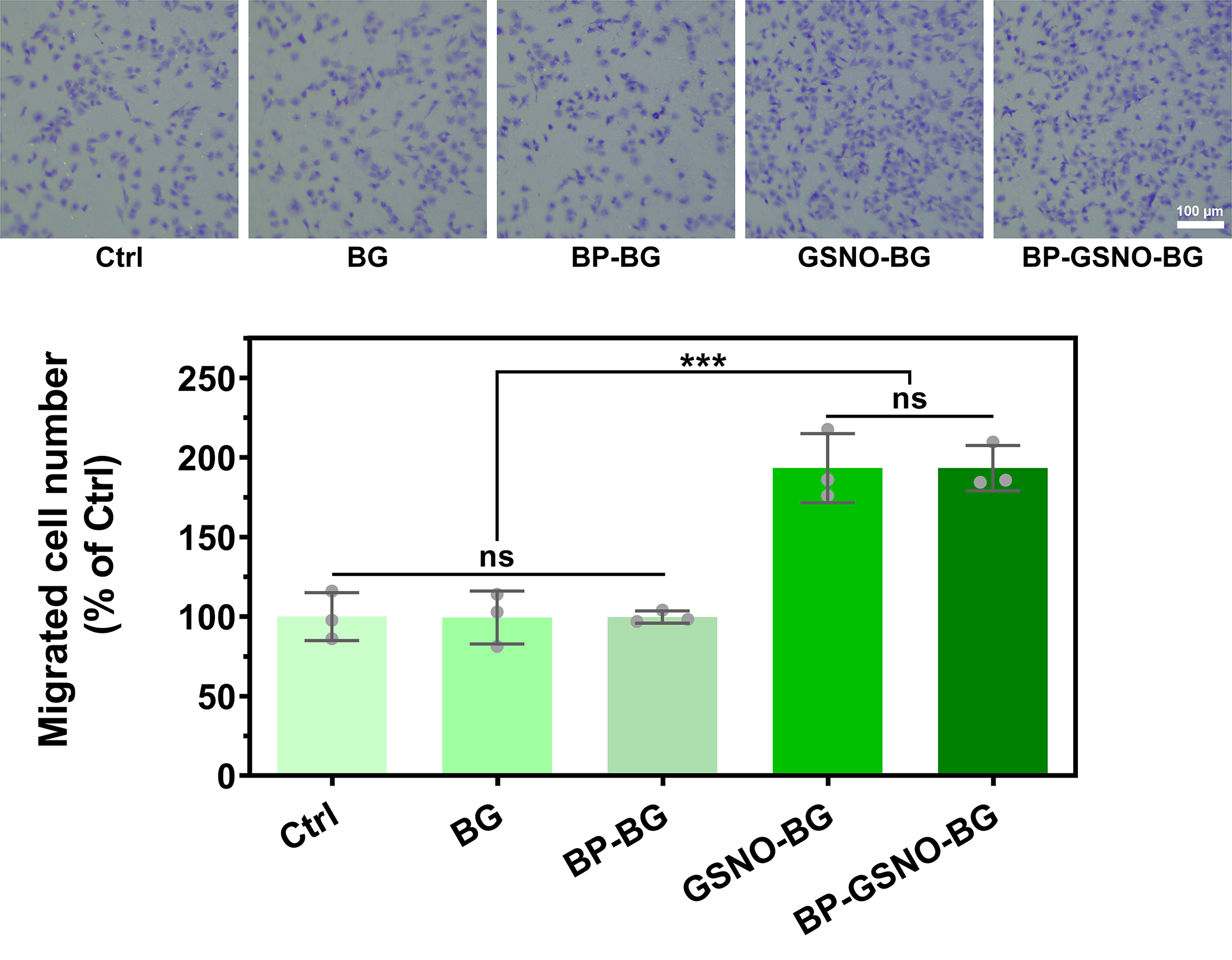


**Figure S12.** *In vitro* assessment of angiogenic effects of the hybrid scaffolds. Transwell test for measuring the migration capability of HUVEC and the corresponding quantification results (Scale bar: 100 µm. Statistics were derived using the one-way analysis of variance. All data were expressed as mean ± standard deviation (SD). n = 3 for each group, ***P < 0.001. Grouping: Ctrl group, HUVECs cultured under normal condition; BG group, HUVECs co-cultured with the BG scaffold; BP-BG group, HUVECs co-cultured with the BP-BG scaffold; GSNO-BG group, HUVECs co-cultured with the GSNO-BG scaffold; BP-GSNO-BG group, HUVECs co-cultured with the BP-GSNO-BG scaffold).


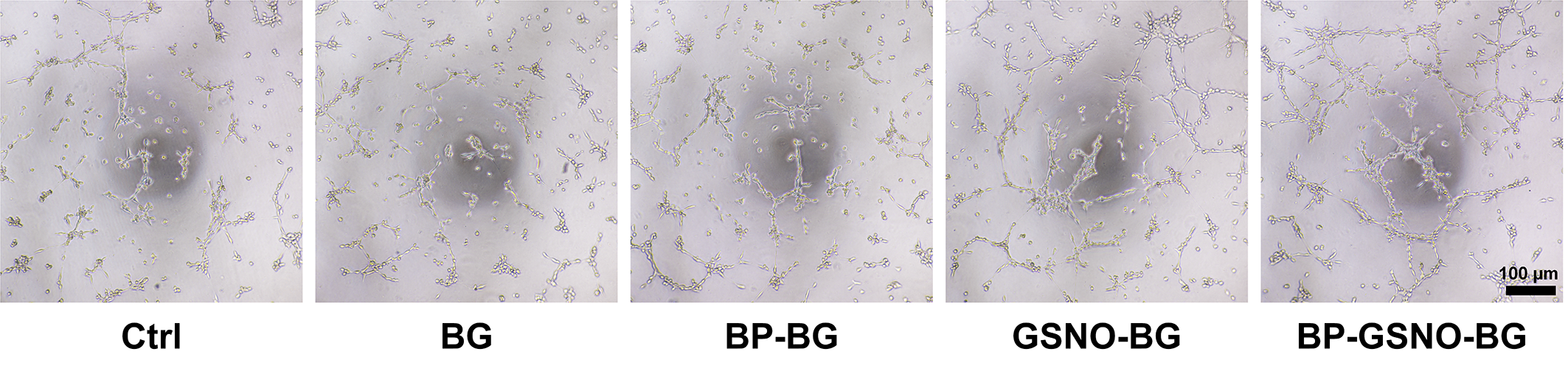


**Figure S13.** *In vitro* assessment of angiogenic effects of the hybrid scaffolds. Tube formation assay by HUVECs incubated with the leaching solution from different scaffolds for 12 hours in Matrigel (Scale bar: 100 µm. Grouping: Ctrl group, HUVECs cultured under normal condition; BG group, HUVECs cultured with the leaching solution from BG scaffold; BP-BG group, HUVECs cultured with the leaching solution from BP-BG scaffold; GSNO-BG group, HUVECs cultured with the leaching solution from GSNO-BG scaffold; BP-GSNO-BG group, HUVECs cultured with the leaching solution from BP-GSNO-BG scaffold).
